# Coupling azo dye degradation and biodiesel production by manganese-dependent peroxidase producing oleaginous yeasts isolated from wood-feeding termite gut symbionts

**DOI:** 10.1186/s13068-021-01906-0

**Published:** 2021-03-08

**Authors:** Sameh Samir Ali, Rania Al-Tohamy, Eleni Koutra, Michael Kornaros, Maha Khalil, Tamer Elsamahy, Mohamed El-Shetehy, Jianzhong Sun

**Affiliations:** 1 Biofuels Institute, School of the Environment and Safety Engineering, Jiangsu University, Xuefu Rd. 301, Zhenjiang, 212013 China; 2 Botany Department, Faculty of Science, Tanta University, Tanta, 31527 Egypt; 3 Laboratory of Biochemical Engineering & Environmental Technology (LBEET), Department of Chemical Engineering, University of Patras, 1 Karatheodori Str., University Campus, 26504 Patras, Greece; 4 INVALOR: Research Infrastructure for Waste Valorization and Sustainable Management, University Campus, 26504 Patras, Greece; 5 Department of Biology, College of Science, Taif University, P.O. Box 11099, Taif, 21944 Saudi Arabia; 6 Department of Plant Science, University of Manitoba, Winnipeg, MB R3T 2N2 Canada

**Keywords:** Ligninases, Textile azo dye, Lignin degradation inhibitors, Wood-feeding termites, Oleaginous yeasts, Biodiesel

## Abstract

**Background:**

Textile industry represents one prevalent activity worldwide, generating large amounts of highly contaminated and rich in azo dyes wastewater, with severe effects on natural ecosystems and public health. However, an effective and environmentally friendly treatment method has not yet been implemented, while concurrently, the increasing demand of modern societies for adequate and sustainable energy supply still remains a global challenge. Under this scope, the purpose of the present study was to isolate promising species of yeasts inhabiting wood-feeding termite guts, for combined azo dyes and textile wastewater bioremediation, along with biodiesel production.

**Results:**

Thirty-eight yeast strains were isolated, molecularly identified and subsequently tested for desired enzymatic activity, lipid accumulation, and tolerance to lignin-derived metabolites. The most promising species were then used for construction of a novel yeast consortium, which was further evaluated for azo dyes degradation, under various culture conditions, dye levels, as well as upon the addition of heavy metals, different carbon and nitrogen sources, and lastly agro-waste as an inexpensive and environmentally friendly substrate alternative. The novel yeast consortium, NYC-1, which was constructed included the manganese-dependent peroxidase producing oleaginous strains *Meyerozyma caribbica*, *Meyerozyma guilliermondii*, *Debaryomyces hansenii*, and *Vanrija humicola*, and showed efficient azo dyes decolorization, which was further enhanced depending on the incubation conditions. Furthermore, enzymatic activity, fatty acid profile and biodiesel properties were thoroughly investigated. Lastly, a dye degradation pathway coupled to biodiesel production was proposed, including the formation of phenol-based products, instead of toxic aromatic amines.

**Conclusion:**

In total, this study might be the first to explore the application of MnP and lipid-accumulating yeasts for coupling dye degradation and biodiesel production.

**Supplementary Information:**

The online version contains supplementary material available at 10.1186/s13068-021-01906-0.

## Background

Currently, most countries worldwide mainly depend on fossil fuel reserves for meeting energy requirements, which leads to various environmental, health and socio-economic issues [1]. Furthermore, the increasing demand of modern societies for adequate energy supply results in an escalating pressure on energy production systems and natural ecosystems, which necessitate a transition in both energy production and consumption [2]. Under this scope, renewable energy sources constitute an environmentally friendly strategy towards climate change mitigation, energy security and sustainable progress, though holistic and careful assessment of renewable energy technologies should incorporate the entire range of factors, including environmental, energetic and socio-economic ones [3]. Biofuels, mainly including bioethanol, biodiesel and biogas, have dramatically increased over the past decades and are currently represented by four generation feedstocks, each one characterized by multiple advantages and drawbacks [4]. However, biotechnological applications need further development to be fully implemented and prevail in the energy sector [5]. Besides energy crisis, the increasing industrialization, urbanization, and population which is estimated to exceed 8.5 billion by 2030, result in the production of billion tons of domestic, agricultural, and industrial wastewater.

Up to 20% of the industrial wastewater pollution emanates from textile industry, which consumes large water volumes along with chemicals during material processing, resulting in highly contaminated and colored effluents [6, 7]. The vast use of synthetic dyes in textile industry can have detrimental effects in case the produced wastewater ends up in the environment, without prior treatment. The quantities which are annually released exceed 10^6^ tons [8], while due to the visible light absorbance they can severely affect microbial and plant photosynthesis [9]. Furthermore, textile wastewater affects pH, organic carbon levels and gas solubility of aquifers [8], while mutagenic and genotoxic effects have been also described [10]. Among a wide range of available dyes, azo dyes, also used in other industries including food and paper processing and cosmetics, are the leading ones. Besides the existence of azo-groups, azo dyes are characterized by complex aromatic structure, high persistence, release of carcinogenic compounds upon degradation, as well as visible contamination even at concentrations up to 50 mg/L [8, 11]. The stability of azo dyes has been previously described, while up to 87.5% could be recovered from soil after a period of two weeks, concurrently affecting the microbial community structure [12]. The available methods for textile wastewater treatment include physical, chemical, and biological processes, with the latter having significant benefits. Physical methods include adsorption, ion exchange, and filtration/coagulation, while chemical methods include ozonisation, Fenton reagent, and photocatalytic reactions [8, 13, 14]. Biological methods are based on the activity of various microorganisms, such as bacteria, yeasts, fungi and microalgae, which degrade textile wastewater and dyes through their enzymatic activity, while the combined synergistic effect of mixed microbial cultures can lead to advanced treatment [11, 15, 16]. Enzymatic degradation of textile wastewater is based on several enzymes, including laccase (Lac), lignin peroxidase (LiP), manganese peroxidase (MnP), and azoreductase among others, while the efficiency of biodegradation depends on the concentration and adaptability of the microorganisms used, O_2_ levels, temperature, as well as dye and organics concentration [7, 17]. In total, biological treatment of dyeing effluents are more environmentally friendly, cost-effective, harmless to living organisms, and generate significantly lower amounts of sludge, compared to other methods [17, 18]. However, biodegradation processes are not yet applied at large scale, thus further research is needed until industrialization [7].

Up to date, the potential of yeasts for azo dyes degradation has not been fully determined, though the decolorization performance of a few species belonging to *Candida*, *Galactomyces*, and *Debaryomyces* genera, as well as *Saccharomyces cerevisiae* have been investigated [11]. A sea mud yeast isolate, determined as *Pichia occidentalis* was effective in Acid Red B degradation, achieving higher than 98% removal out of 50 mg/L, during a 16 h-cultivation [19]. Similarly, higher than 92% removal of the Acid Scarlet GR, at a concentration of 100 mg/L, was achieved by the salt-tolerant *Galactomyces geotrichum* yeast, while the derived metabolites were non-toxic upon plant application [20]. Furthermore, the termite isolate *Sterigmatomyces halophilus* could effectively metabolize up to 1.5 g/L of the azo dye Reactive Black 5 [13, 14]. Besides monocultures, however, as previously described in case of bacterial consortia [8], co-cultivation of microorganisms with desired characteristics can increase process effectiveness. Such enhanced performance has been observed in case of a constructed yeast consortium including three yeast isolates from termite guts, resulting in high bio-degradation efficiency of azo dyes, even in case of dye mixtures which could be decolorized up to about 81% [18]. Interestingly, azo dye catabolism could be combined with other valuable features of yeasts, such as lipid accumulation, to concurrently add value to the treated substrates through production of biofuels. That was the purpose of Yaguchi et al. [21] who have recently tested 36 oil-producing yeasts for their ability to degrade metabolites of lignin and additionally be utilized for lipid production. However, similar reports are rather scarce, and almost totally unexplored in case of yeast termite symbionts. Under this scope, the purpose of this study was to isolate promising species of yeasts inhabiting wood-feeding termite guts, for combined azo dyes and textile wastewater bioremediation, along with biodiesel production. For this purpose, 38 strains in total were isolated, followed by molecular identification. Yeast isolates were subsequently tested for the desired enzymatic (MnP) activity, lipid accumulation, and tolerance to lignin-derived metabolites. The most promising species were then used for construction of a novel yeast consortium, which was further evaluated for azo dyes degradation. Furthermore, decolorization efficiency was investigated in terms of culture conditions, azo dye levels, as well as upon the addition of heavy metals, various carbon and nitrogen sources, and lastly agro-waste as an inexpensive and environmentally friendly substrate alternative. Studies on utilization of lignin and/or lignin-like dyes by oleaginous yeasts hold much promise for achieving overall efficiency and sustainable utilization of lignocellulosic biomass and textile azo dyes for biofuel production.

## Results

### Screening of MnP-producing yeasts isolated from termite gut symbionts

MnP is an important ligninolytic enzyme responsible for the mineralization of several textile dyes of different structures, such as azo dyes‚ through various processes, including redox reaction, which oxidizes phenolic compounds with conversion of Mn^2+^ to Mn^3+^ [22]. Compared to filamentous fungi, dye decolorization by yeasts based on MnP production is scarce and still fragmentary [23, 24]. Several yeast species employed for the decolorization of textile dyes through MnP production, include *Candida tropicalis*, *Candida oleophyla*, *Pichia occidentalis*, and *Debaryomyces polymorphus* [25–27]. In this study, 38 yeast isolates were positive for MnP production in terms of their clear zone formation on the YPD agar plates containing o-dianisidine dihydrochloride (a precursor of many azo dyes). These yeasts were isolated from guts of the wood-feeding termite, *Reticulitermes chinenesis* and *Coptotermes formosanus* and were mainly found to belong to the species of *Candida* sp., *Pichia* sp., *Wickerhamomyces* sp., *Cyberlindnera* sp., *Candida silvanorum*, *Candida stauntonica*, *Candida tropicalis*, *Cyberlindnera bimundalis*, *Debaryomyces hansenii*, *Fellozyma inositophila*, *Meyerozyma guilliermondii*, *Meyerozyma caribbica*, *Yarrowia* sp., and *Vanrija humicola*. However, to the best of our knowledge, there are some novel yeast species isolated from *R. chinenesis* and *C. formosanus* that were first to be confirmed with such feature, including *Starmera dryadoides*, *Sterigmatomyces halophilus*, *Candida gotoi*, and *Hamamotoa lignophila*. Recently, there has been an increase in the number of studies on the gut symbionts of xylophagous insects like termites, especially termite digestome, both termites and their gut symbionts, which have many potential bioenergy applications that need careful consideration [28, 29]. In contrast to previous studies on lignin breakdown, which gave no convincing evidence of microbial degradation of lignin in the termite intestinal tract [30], recent studies reported that the paunch of termites harbors a significant number of lignin-degrading yeasts [31–34]. Schäfer et al. [35] reported that yeasts were ranged between 10^7^ and 5 × 10^8^ cells/mL in the gut of *Zootermopsis angusticollis* and *Neotermes castaneus*. These yeasts were mainly represented by *Candida*, *Pichia*, and *Debaromyces*. On the other hand, transcriptomic analysis of termite host tissues revealed that the presence of lignin-degrading fungi in termite guts enable such symbionts for nearly complete degradation of lignocellulose [33].

After flooding the plates with H_2_O_2_, the reddish-brown color change was observed in around 31 yeast colonies, which is positively correlated with the production of MnP enzyme. Based on the ratio of the diameter of the clear zone to the yeast colony, 22 isolates showed a ratio higher than 5 (data not shown). In this study, nine isolates exhibited the highest extracellular MnP activity (ranged between 23 and 27 U/mL) after 120 h of incubation at 25 °C in the basal medium supplemented with o-dianisidine dihydrochloride. The pH of the culture medium was adjusted to 5.1. These peroxidase-producing yeast isolates were identified as *Cyberlindnera* sp. strain SSA1583 (KX791370), *Candida stauntonica* SSA1653 (KY172950), *Meyerozyma caribbica* strain SSA1654 (KY172951), *Meyerozyma guilliermondii* strain SSA1547 (KX907633), *Yarrowia* sp. strain SSA1642 (KX907706), *Candida silvanorum* strain SSA1643 (KX907707), *Sterigmatomyces halophilus* strain SSA1655 (KY172952), *Debaryomyces hansenii* strain SSA1502 (KX791388), and *Sugiyamaella* sp. nov. strain SSA1650 (KX907714). The MnP enzyme produced by *Geotrichum* sp. has proved to be constitutive, achieving maximum activity within 5 days, while this yeast species showed higher MnP titers in RB5-amended medium [36]. On the other hand, MnP produced by *Debaryomyces polymorphus* and *Candida tropicalis* proved to be more inducible in a medium supplemented with dye, compared to the control [27]. Due to its broad substrate specificity, MnP is capable of destructing or transforming synthetic dyes into innocuous end products [37]. In addition, yeasts have potential for waste bioremediation. Therefore, screening of yeasts for ligninolytic peroxidases’ activity, is of high demand since such producers have high potential for biodegradation of hazardous organic pollutants (e.g. textile azo dyes), and other biotechnological applications, such as biodiesel production.

### Lipid production and microbial characterization

Twenty-two MnP-producing yeast isolates were qualitatively examined for the production of neutral lipids using Sudan black B staining. Of these yeasts, 14 isolates were stained blue and were further evaluated by Nile red staining. Sudan black B is lipophilic stain that cannot determine different lipid classes compared with Nile red, which is used to quantify intracellular neutral lipid by fluorescence spectroscopy [38]. On the other hand, seven MnP-producing yeast isolates (SR-4, SR-11, SR-18, SR-22, SR-27, SR-32 and SR-35) showed a high yellow-gold coloration upon Nile red staining and fluorometric examination.

**Fig. 1:**
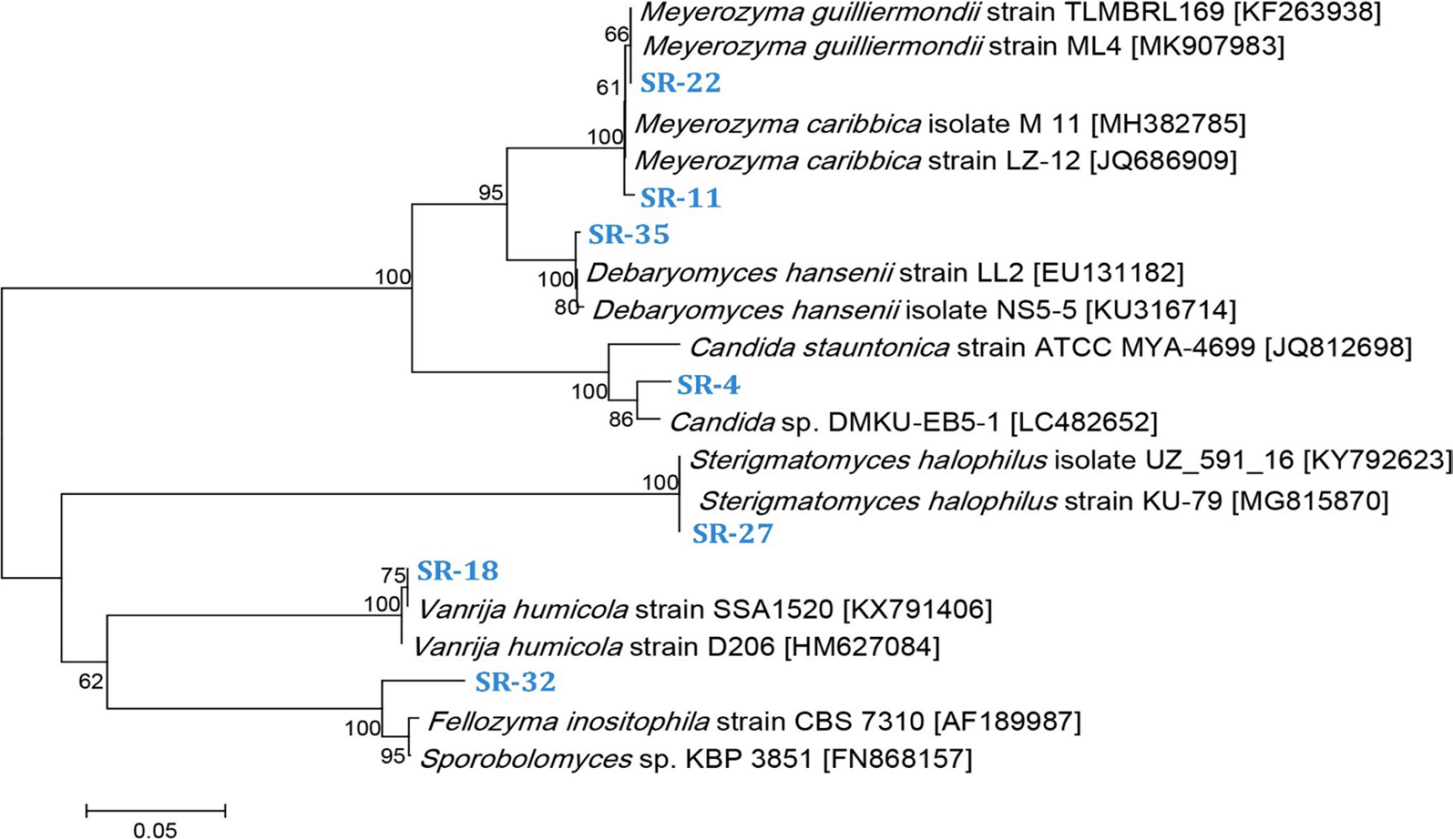
A Neighbor-Joining phylogenetic tree of manganese peroxidase-producing/lipid-accumulating yeast strains with closely related taxa in NCBI database, based on the D1/D2 domain region. The horizontal bar at the bottom represents evolutionary distance as 0.05 changes per nucleotide position. Numbers next to the tree branches represent the bootstrap values as percentages of 1000 replicates

The relationship of the selected seven yeast isolates with their nearest phylogenetic relatives was also studied. These isolates proved to be closely related to the following yeast genera: *Candida*, *Meyerozyma*, *Fellozyma*, *Vanrija*, and *Sterigmatomyces*. The phylogenetic tree revealed that the yeast isolates SR-4, SR-11, SR-22 and SR-35 belong to Ascomycota phylum and were subsequently named as *Candida stauntonica* strain SSA1653 (KY172950), *Meyerozyma caribbica* [former *Candida fermentati*] strain SSA1654 (KY172951), *Meyerozyma guilliermondii* [former *Pichia guilliermondii*] strain SSA1547 (KX907633), and *Debaryomyces hansenii* strain SSA1502 (KX791388), respectively. The later four Ascomycetous yeast strains showed 95.40, 99.30, 100.00 and 99.49% identity to *Candida stauntonica* strain ATCC MYA-4699 (JQ812698), *Meyerozyma caribbica* strain LZ-12 (JQ686909), *Meyerozyma guilliermondii* strain ML4 (MK907983), and *Debaryomyces hansenii* strain LL2 (EU131182), respectively (Fig. 1). The other three yeast isolates SR-18, SR-27 and SR-32 belong to Basidiomycota phylum and were denoted as *Vanrija humicola* [former *Cryptococcus humicola*] strain SSA1514 (KX791400), *Sterigmatomyces halophilus* strain SSA1655 (KY172952), and *Fellozyma inositophila* strain SSA1579 (KX791364), respectively. The later three Basidiomycetous yeast strains showed 99.34, 99.67 and 95.44% identity to *Vanrija humicola* strain SSA1520 (KX791406), *Sterigmatomyces halophilus* strain KU-79 (MG815870), and *Fellozyma inositophila* strain CBS 7310 (AF189987), respectively (Fig. 1).

Insects are common vectors of yeasts in nature and screening of yeasts from insect guts leads to the discovery of numerous novel yeast species with various potential biotechnological applications [13, 29, 39–41]. Termites are the most well-studied group of social insects with regard to the processing and fate of dietary carbon, while they also represent efficient lignocellulose mineralizers, capable of removing up to 99% and 87% of cellulose and hemicellulose, respectively [28]. Investigation of termites and further application of their gut-inhabiting yeasts in bioremediation and biorefinery industries is still in its infancy. To our knowledge, there have been no reports up to date on the isolation of the oleaginous yeast species *Candida stauntonica* from wood-feeding termites. In this study, *Candida stauntonica* strain SSA1653, was isolated from *Coptotermes formosanus* for the first time. *Coptotermes formosanus* is a typical lower wood-feeding termite that evolved a sophisticated lignocellulolytic system to effectively metabolize woody materials [33, 42]. Recently, *Candida stauntonica* was successfully isolated from the gut of adult beetle insects, which were collected from trunks and rotten wood at Henan Province, China [40]. On the other hand, the lipid-accumulating yeast strains *Meyerozyma caribbica* SSA1654 and *Meyerozyma guilliermondii* SSA1547, were successfully isolated from *Coptotermes formosanus* and *Reticulitermes chinenesis*, respectively. As reported previously, *Meyerozyma caribbica* and *Meyerozyma guilliermondii* are two closely related yeast species, belonging to the *Meyerozyma guilliermondii* species complex [43]. Several uses have been reported for *Meyerozyma guilliermondii*, including riboflavin production, enzyme production, bioconversion of xylose into xylitol, as well as biofuel production [44–46]. Besides, *Meyerozyma caribbica* strain DMKURK258 and *Meyerozyma guilliermondii* strain B1281A have been previously reported as new potential oleaginous yeasts intended for biodiesel production [47]. Moreover, yeasts of the *Meyerozyma guilliermondii* clade possess a wide range of anti-microbial activities against bacteria, fungi, and protozoa and can be found in insect hosts [47], opening the possibility of envisioning similar anti-microbial activity approaches.

In this study, the oleaginous yeast strain *Debaryomyces hansenii* SSA1502, was successfully isolated from the wood-feeding termite species *Coptotermes formosanus*. Ratledge and Tan [48] reported that *Debaryomyces hansenii* is an oil-producing yeast with high potential for the biotechnological production of both natural and artificial products. It is able to tolerate several toxins and chlorine dioxide (ClO_2_), as a powerful biocide [49]. Furthermore, the ability of *Debaryomyces hansenii* to tolerate extreme stress could be highly advantageous in the implementation of low-cost fermentation processes [50]. It has been reported that *Debaryomyces hansenii* efficiently produces xylitol from D-xylose in wood hydrolysates, generating high ratios of xylitol to ethanol. Moreover, *Debaryomyces hansenii* synthesizes several enzymes of industrial importance, such as inulinases, esterases and β-glucosidases [50].

The lipid-accumulating *Vanrija humicola* (former *Cryptococcus humicola*) strain SSA1514 was isolated from *Reticulitermes chinenesis*. Souza et al. [51] reported the role of *Cryptococcus humicola* CCMA 0346 (isolated from soil) for lipid production. Similarly, *Cryptococcus humicola* strain UCDFST 10-1004 efficiently converted lignocellulosic sugars into neutral lipids (TAG) for biodiesel production [52]. Although the medical potential of several *Sterigmatomyces* strains has been previously reported, the newly isolated and unique yeast strain *Sterigmatomyces halophilus* SSA-1575, was inhabiting the gut system of *Reticulitermes chinenesis* and it showed great potential for dye decolorization and detoxification, as reported previously [13, 14]. However, to the best of our knowledge, there have been no current reports on lipid accumulation belonging to this yeast species. In this study, the lipid-accumulating yeast strains *Sterigmatomyces halophilus* SSA1655 and *Fellozyma inositophila* SSA1579 were successfully isolated from *Reticulitermes chinenesis*.

As Nile red molecules may also non-specifically bind to specific non-lipid cellular compartments [53], lipid content quantification was necessary to confirm the yeasts’ oleaginous nature. Therefore, the selected yeast strains were cultivated into the same N-limited medium followed by lipid extraction. Biomass and lipid production were determined for the seven selected yeast strains (SSA1653, SSA1654, SSA1547, SSA1502, SSA1514, SSA1655, and SSA1579), after 5 days of incubation at 25 °C in the N-limited glucose-based medium containing 0.5 g/L o-dianisidine dihydrochloride as a precursor of many azo dyes. The parameters characterizing biomass and lipid production by the oleaginous yeast strains were depicted in Table 1. The highest values of biomass (13.28 ± 0.57 to 15.78 ± 0.45 g/L), lipid production (4.51 ± 0.88 to 7.10 ± 0.85 g/L), lipid content (33.96 ± 5.72 to 47.25 ± 1.84%, w/w), *Y*_*L/x*_ (0.340 ± 0.07 to 0.473 ± 0.03 g/g), *Y*_*x/s*_ (0.40 ± 0.005 to 0.50 ± 0.03 g/g), *Y*_*L/s*_ (0.17 ± 0.02 to 0.23 ± 0.01 g/g), *Q*_*x*_ (0.10 ± 0.007 to 0.15 ± 0.004 g/L/h) and *Q*_*L*_ (0.04 ± 0.005 to 0.08 ± 0.003 g/L/h) were reached by the oleaginous yeast strains *Meyerozyma caribbica* SSA1654, *Meyerozyma guilliermondii* SSA1547, *Debaryomyces hansenii* SSA1502, and *Vanrija humicola* SSA1514 (Table 1). Our findings are in agreement with those reported by Maza et al. [54] who found that *Rhodotorula glutinis* R4, *Rhodotorula toruloides* Y-1091, *Rhodotorula toruloides* Y-6987, and *Rhodotorula toruloides* Y-6985 revealed the highest values of biomass (14–16 g/L), lipid production (6.8–7.3 g/L), lipid content (43.9–48.9%, w/w), *Y*_*L/x*_ (0.45–0.50 g/g), *Y*_*x/s*_ (0.40–0.47 g/g), *Y*_*L/s*_ (0.18–0.22 g/g), *Q*_*x*_ (0.119–0.134 g/L/h) and *Q*_*L*_ (0.057–0.061 g/L/h). On the other hand, *Candida viswanathii* Y-E4 showed the highest lipid yield (3.55 g/L). However, *Rhodotorula babjevae* Y-SL7 showed the highest lipid content (39.2%) and lipid yield by consumed glucose (Y_L/s_, 0.1 g/g) as reported by Ayadi et al. [55]. As depicted in Table 2, various other oleaginous yeast genera, such as *Cryptococcus*, *Cyberlindera*, *Candida*, *Cystobasidium*, *Rhodotorula*, *Rhodosporidium*, and *Yarrowia*, have been reported to have comparable values of biomass, lipid content and lipid productivity in case of glucose or biomass waste used as a carbon source. *Meyerozyma caribbica* SSA1654, isolated from termite gut symbionts, showed higher lipid productivity and comparable with *Rhodotorula toruloides* Y-1091 [54], *Rhodotorula glutinis* R4 [54], *Candida viswanathii* Y-E4A [56], *Cystobasidium oligophagum* JRC1 [57], *Rhodosporidium kratochvilovae* HIMPA1 [58], *Cryptococcus curvatus* [59]*,* and *Rhodosporidium toruloides* Y4 [60].

**Table 1:** Biomass and lipid parameters produced by the oleaginous MnP-producing yeast strains isolated from termite gut symbionts

Yeast strains*	Biomass (g/L)	Lipid (g/L)	Lipid content (%, w/w)	*Y*_*L/x*_ (g/g)	*Y*_*x/s*_ (g/g)	*Y*_*L/s*_ (g/g)	*Q*_*x*_ (g/L/h)	*Q*_*L*_ (g/L/h)
*Meyerozyma caribbica* SSA1654	14.71 ± 0.38^a^	6.95 ± 1.77^a^	47.25 ± 1.84^a^	0.473 ± 0.03^a^	0.50 ± 0.03^a,c^	0.22 ± 0.03^a^	0.14 ± 0.004^a^	0.08 ± 0.003^a.c^
*Vanrija humicola* SSA1514	15.23 ± 0.09^a,b^	6.62 ± 1.13^a^	43.46 ± 5.34^a,b^	0.435 ± 0.08^a^	0.47 ± 0.008^a^	0.23 ± 0.04^a^	0.15 ± 0.009^a^	0.05 ± 0.007
*Meyerozyma guilliermondii* SSA1547	15.78 ± 0.45^a,b^	7.10 ± 0.85^a,c^	44.74 ± 6.09^a,b^	0.447 ± 0.07^a^	0.48 ± 0.009^a^	0.23 ± 0.01^a^	0.15 ± 0.004^a^	0.06 ± 0.005^a^
*Debaryomyces hansenii* SSA1502	13.28 ± 0.57	4.51 ± 0.88	33.96 ± 5.72	0.340 ± 0.07	0.40 ± 0.005	0.17 ± 0.02	0.10 ± 0.007	0.04 ± 0.005

The values are mean of three independent experiments. Values followed by the same letters showed insignificant difference; *Yeasts were grown in the N-limited glucose-based medium at 20 °C during 120 h; ^a^Significant reference towards *Debaryomyces hansenii* SSA1502; ^b^Significant reference towards *Meyerozyma caribbica* SSA1654; ^c^Significant reference towards *Vanrija humicola* SSA1514

**Table 2:** Lipid production by different oleaginous yeasts reported in literature

Oleaginous yeast	Source	Carbon source	Cultivation mode	Biomass production (g/L)	Lipid content (%)	Lipid productivity (g/L/h)	Reference
*Rhodotorula toruloides* Y-1091	Culture collection	Glucose	Batch	14.90	48.93	0.06	Maza et al. [54]
*Rhodotorula glutinis* R4	Soil	Glucose	Batch	14.325	47.24	0.057	Maza et al. [54]
*Candida viswanathii* Y-E4	Soil	Glucose	Batch	13.62	25.33	0.029	Ayadi et al. [56]
*Cystobasidium oligophagum* JRC1	Soil	Glucose	Batch	12.34	39.44	0.029	Vyas and Chhabra [57]
*Rhodosporidium kratochvilovae* HIMPA1	Soil	Glucose	Batch	14.46	41.92	0.037	Patel et al. [58]
*Cyberlindera fabianii* Y-B14	Olive pomace	Glucose	Batch	6.01	25.58	ND	Ayadi et al. [55]
*Candida tropicalis* Y-L2	Palm juice	Glucose	Batch	6.23	22.10	ND	Ayadi et al. [55]
*Yarrowia lipolytica* Y-RC7	Cheese wastewater	Glucose	Batch	6.81	26.63	ND	Ayadi et al. [55]
*Cryptococcus curvatus*	Culture collection	Sorghum bagasse	Batch	6.0	NA	0.036	Liang et al. [59]
*Rhodosporidium toruloides* Y4	NA	Corn stover	Batch	14.2	51.8	0.035	Xie et al. [60]
*Meyerozyma caribbica* SSA1654	Termite gut	Glucose	Batch	14.71	47.25	0.08	This study
*Meyerozyma guilliermondii* SSA1547	Termite gut	Glucose	Batch	15.78	44.74	0.06	This study
*Vanrija humicola* SSA1514	Termite gut	Glucose	Batch	15.23	43.46	0.05	This study
*Debaryomyces hansenii* SSA1502	Termite gut	Glucose	Batch	13.28	33.96	0.04	This study

*NA* not available, *ND* not determined

To produce biodiesel, selection of yeasts with oleaginous nature is a vital stage, followed by the implementation of the appropriate cultivation system for growth and lipid accumulation. The produced biomass was harvested, proceeding with the extraction of lipids from oleaginous yeasts, followed by the transesterification into fatty acid methyl esters (FAMEs) [61]. In that context, four strains (*Meyerozyma caribbica* SSA1654, *Meyerozyma guilliermondii* SSA1547, *Debaryomyces hansenii* SSA1502, and *Vanrija humicola* SSA1514) appeared as promising oleaginous yeasts, with accumulated lipids between 33.96 ± 5.72 and 47.25 ± 1.84% (w/w) (Table 1), when compared with the commercial oleaginous yeast strain *Yarrowia lipolytica* W29 which produces 1.7 g/L corresponding to 29% of lipid content [55]. Ascomycetes, such as *Candida tropicalis* and *Yarrowia lipolytica*, are the best lipid-producing yeasts [62]. In this study, the highest percentage of lipid accumulation was recorded for the first time for *Meyerozyma caribbica* strain SSA1654, isolated from wood-feeding termite guts, since it showed a remarkable ability of lipid accumulation up to 47.25 ± 1.84% (w/w). Maza et al. [54] reported that among nine oleaginous yeast strains evaluated for lipid production, *Rhodotorula glutinis* R4 showed the highest lipid production (7 g/L; 47% w/w) with efficient growth and glucose consumption. The highest lipid yield (39.2%) was achieved by *Rhodotorula babjevae* Y-SL7, while the highest lipid content (3.55 g/L) was achieved by *Candida viswanathii* Y-E4, since xylose was used as the carbon source [55].

### Yeast growth, glucose residual and nitrogen assimilation with respect to lipid production

**Fig. 2:**
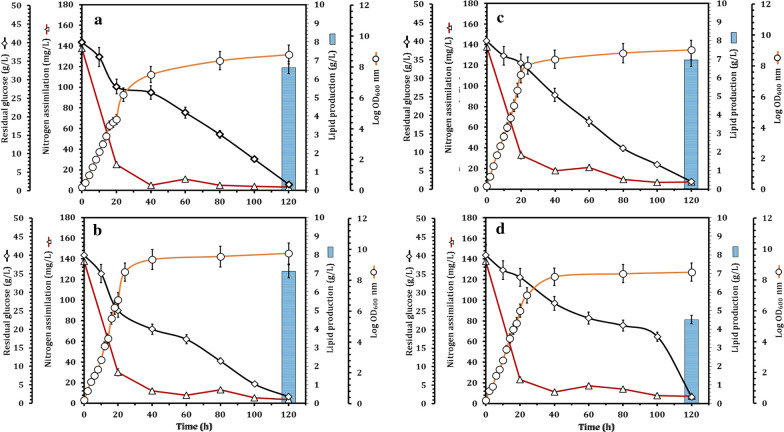
Oleaginous yeast strains; *V. humicola* SSA1514 (**a**), *M. guilliermondii* SSA1547 (**b**), *M. caribbica* SSA1654 (**c**), and *D. hansenii* SSA1502 (**d**) showing their growth (log OD_600_ nm), residual glucose, nitrogen assimilation in association with lipid production, after 5 days of cultivation in N-limited glucose-based medium. Values are mean of three independent replicates and the standard deviation is indicated by error bars

The oleaginous yeast strains (*Meyerozyma caribbica* SSA1654, *Meyerozyma guilliermondii* SSA1547, *Debaryomyces hansenii* SSA1502, and *Vanrija humicola* SSA1514) were grown in liquid N-limited glucose-based medium supplemented with o-dianisidine dihydrochloride. The inoculated flasks were incubated at 25 °C; meanwhile, all yeasts were characterized using the classic growth curves for 120 h. Clearly, the oleaginous yeast strains presented the typical growth curves with logarithmic and stationary phases (Fig. 2). The cells entered stationary phase and thus no substantial OD increase was observed after the depletion of nitrogen. However, glucose consumption continued until the end of experiments at 120 h. Nitrogen was almost completely depleted after 24 h of growth, with assimilation reaching up to 96%, while residual glucose was less than 15% inducing the onset of stationary growth phase. At the end of the incubation time (120 h), 3.9–5.2% of glucose remained in the cultures of the oleaginous yeast strains SSA1654, SSA1547, SSA1502, and SSA1514, respectively (Fig. 2).

On the other hand, nitrogen was sharply depleted within 24 h, achieving 86–96.4% removal, and then remained constant for all yeast strains until the end of the incubation time (Fig. 2). In the presence of excess carbon source, yeasts accumulate it in the form of TAG, comparing with bacteria that metabolize carbon into polysaccharides and polyhydroxyalkanoates [61]. Such yeasts can produce lipids by metabolizing different carbon sources [63]. As previously reported, oleaginous yeasts can accumulate lipids which are similar to vegetable oils [64].

Among the four oleaginous yeast strains, *Meyerozyma caribbica* SSA1654 reached the highest lipid production (47.25 ± 1.84%, w/w) with an initial nitrogen assimilation of 137.5 mg/L and 40 g/L of glucose (Fig. 2c), resulting in a glucose-rich culture medium, though poor in nitrogen. As a result of such an unbalanced metabolism, the oleaginous yeasts can continue glucose uptake, which is further converted into TAG and stored as energy reserves. Evans and Ratledge [65] reported three growth phases of oleaginous yeasts under nutrient limitation conditions. Of those, exponential phase exhibits rapid proliferation of yeast cells. In the lipid accumulation phase, yeast cells show minimum growth due to nutrient limitation (e.g. nitrogen, sulfur and phosphorus), while in the stationary phase, catabolic breakdown of lipid occurs resulting in low lipid content. However, upon the depletion of nitrogen, the intracellular concentration of NH_4_^+^ was rapidly increased by oleaginous yeasts since the adenine-mono-phosphate (AMP) was broken down by AMP-deaminase to give inosine-mono-phosphate (IMP) and NH_4_^+^ (as a temporary nitrogen source for the yeasts to continue their growth) [66]. Furthermore, *Meyerozyma caribbica* SSA1654 is a cold-tolerant oleaginous yeast and it has been reported that cold-tolerant or cold-adapted yeasts are excellent candidates for microbial lipids production [67]. These yeasts have a high content of mono-unsaturated fatty acid (MUFA), mainly oleic acid (C18:1), and are potentially valuable for biodiesel production under cold conditions, e.g. during the winter period [68].

### Characterization of extracted lipids

Fatty acid composition of microbial lipids, determined as FAME profile, greatly affects biodiesel properties. Under this scope, TAG synthesized by *Meyerozyma caribbica* SSA1654 was assessed. Table 3 depicts the fatty acid composition of *Meyerozyma caribbica* SSA1654, which was mainly rich in long-chain fatty acids (C16 and C18), compared to olive oil. The saturated fatty acid (SFA), MUFA and poly-unsaturated fatty acid (PUFA) represent over 95% of total fatty acids, which are similar to that of vegetable oils, containing predominantly SFA or MUFA with 16 and 18 carbon atoms [54, 55, 69], suggesting that *Meyerozyma caribbica* SSA1654 represents a potential candidate for third-generation biodiesel production. The FAME profile of *Meyerozyma caribbica* SSA1654 revealed that fatty acids with C18 were the most abundant, representing 76.22% (Table 3). Rossi et al. [67] reported that fatty acids with C18 chain length appear as an adaptive character of oleaginous yeasts which are grown under cold conditions. Such habitats may be necessary for the elongation of fatty acids beyond C16 by introducing additional double bonds via Δ12 and Δ15 denaturases [67]. On the other hand, the content of oleic acid (C18:1) in *Meyerozyma caribbica* SSA1654 was found to be 60.73%, while palmitic acid (C16:0) and linoleic acid (C18:2) contents were 17.34% and 9.56% of the total fatty acids, respectively (Table 3). The fatty acid composition of *Meyerozyma caribbica* SSA1654 is in accordance with that of other oleaginous yeast previously reported [15, 54]. Total UFA of *Meyerozyma caribbica* SSA1654 were predominant (75.96%), while total SFA, represented 19.2% (Table 3), in agreement with previous results [54]. In total, the ratio of UFA and SFA of the produced FAME is critical for evaluating biodiesel quality [69], therefore, *Meyerozyma caribbica* SSA1654 is propitious for biodiesel synthesis.

**Table 3:** Fatty acid profile produced by oleaginous yeast strain SSA16541, comparing with common vegetable oil

	Relative fatty acid content (%, w/w)	
	*Meyerozyma caribbica* strain SSA1654	Olive oil
Myristic acid (C14:0)	1.78	0.0
Pentadecanoic acid (C15:0)	0.59	Not determined
Palmitic acid (C16:0)	17.34	11.6
Palmitoleic acid (C16:1)	1.60	1.0
Stearic acid (C18:0)	1.86	3.1
Oleic acid (C18:1)	60.73	75.0
Linoleic acid (C18:2)	9.56	7.8
Linolenic acid (C18:3)	4.07	0.1
Total C16	18.94	12.6
Total C18	76.22	86.0
Total saturated fatty acid	19.2	14.7
Total mono-unsaturated fatty acid	62.33	76.0
Total poly-unsaturated fatty acid	13.63	7.9
Reference	This study	Ramos et al. [69]

*Meyerozyma caribbica* SSA1654 was grown in the N-limited medium supplemented with 40 g/L of glucose at 20 °C during 120 h; The values are mean of three independent experiments

### Assessment of biodiesel quality produced by *Meyerozyma caribbica* SSA1654

The main physicochemical properties of biodiesel produced by *Meyerozyma caribbica* SSA1654, including cetane number (CN), iodine value (IV), saponification value (SV), kinematic viscosity (ν), density (ρ), oxidative stability (OS), long-chain saturation factor (LCSF), and degree of unsaturation (DU) were estimated (Table 4). The obtained values were compared with those produced from olive oil [69], and the international biodiesel standard EN 14214 [70]. It has been reported that the ratio of UFA and SFA must be optimum to enhance biodiesel quality [69]. In this study, the CN of the biodiesel produced by *Meyerozyma caribbica* SSA1654 was found to be 52.32. This value met the CN values of EN 14214 (CN ≥ 51). Patel et al. [61] reported that high CN value ensures less pollutant emissions and better combustion, accordingly, producing efficient engine performance. The IV value of the biodiesel produced by *Meyerozyma caribbica* SSA1654 was 92.37 g I_2_/100 g and it met the value established by EN 14214 (Table 4). As reported previously [71], IV value is greatly affected by the UFA degree and it ensures the oxidation stability of the fuel. The quality of biodiesel also depends on the ν and ρ values. As shown in Table 4, the ν value of biodiesel produced by *Meyerozyma caribbica* SSA1654 (3.98 mm^2^/s) met that estimated from the standard biodiesel EN 14214, which ranged between 3.5 and 5.0 mm^2^/s. Maza et al. [54] reported that the appropriate *ν* value ensures adequate biodiesel supply at various operating temperatures. Also, the *ρ* value of the biodiesel produced by *Meyerozyma caribbica* SSA1654 was found to be 0.88 g/cm^3^ and it met the ρ value established by EN 14214 (Table 4). The *ρ* value of a biodiesel determines its net energy content [61]. The SV, OS, LCSF and DU values of the biodiesel produced by *Meyerozyma caribbica* SSA1654 were 199.4 mg KOH, 8.30 h, 3.34% wt., and 96.85% wt., respectively. These results are in accordance with previous findings [54, 61]. The biodiesel produced by *Meyerozyma caribbica* SSA1654 showed a C18:3 content of 5.72%, which met the content of biodiesel established by EN 14214 (≤ 12). Therefore, the results demonstrated the potential of the MnP-producing/TAG-accumulating *Meyerozyma caribbica* SSA1654 as a novel candidate for biodiesel production.

**Table 4:** Biodiesel properties produced by *Meyerozyma caribbica* SSA1654 in comparison with those of olive oil and the international biodiesel standard EN 14214

Biodiesel properties	*Meyerozyma caribbica* SSA1654	Olive oil	EN 14214
Long chain saturated factor (%, wt)	3.34	4.2	Not specified
Kinematic viscosity (mm^2^/s)	3.98	4.5	3.5–5.0
Iodine value (g I_2_/100 g)	92.37	84.0	≤ 120
Cetane number	52.32	57.0	≥ 51
Saponification value (mg KOH)	199.4	Not determined	Not specified
Linolenic acid (C18:3)	4.07	0.6	≤ 12
Degree of unsaturation (%, wt)	95.85	92.7	Not specified
Density (g/cm^3^)	0.88	Not specified	0.86–0.9
Oxidation stability (h)	8.3	3.3	≥ 6
Reference	This study	Ramos et al. [69]	CEN [70]

### Enzyme activity

The activities of β-glucosidase, CMCase, xylanase and lipase produced by *Meyerozyma caribbica* SSA1654, were determined. The time course of β-glucosidase revealed that all yeast strains achieved the maximum production after three days of incubation with the highest activity of 0.38 ± 0.06 U/mg by *Meyerozyma caribbica* SSA1654. The activities of CMCase and xylanase initiated the first day of cultivation and reached their maximum production after three and four days, respectively. CMCase and xylanase reached their maximum activities of 0.17 ± 0.03 and 5.8 ± 0.9 U/mg by *Meyerozyma caribbica* SSA1654, respectively. The activities of β-glucosidase, CMCase, and xylanase of the given MnP-producing oleaginous yeast strains were comparable with the recently reported oleaginous yeasts. The oleaginous yeast strain *Trichosporon asahii* Y-SL1 reached the maximum CMCase (0.11 U/mL) and β-glucosidase (0.55 U/mL) after three and four days of culture [55]. *Pseudozyma brasiliensis* showed the highest β-glucosidase of 0.14 IU/mL [72]. *Cystobasidium oligophagum* JRC1 exhibited the highest specific enzyme activity for β-glucosidase (0.98 ± 0.04 IU/mg) and CMCase (2.27 ± 0.007 IU/mg) with enzymatic activities of 0.03 ± 0.01 and 0.072 ± 0.01 IU/mL, respectively [57]. On the other hand, the production rate of lipase activity revealed 55.3 ± 5.4 U/mL for *Meyerozyma caribbica* SSA1654. The production rate of lipase activity varied from 16.6 U/mL for *Candida viswanathii* Y-E4 to 50 U/mL for *Yarrowia lipolytica* Y-D1P [56]. *Cystobasidium oligophagum* JRC1 revealed the highest lipase activity of 0.14 ± 0.009 IU/mL and specific activity of 2.88 ± 0.17 IU/mg after 84 h of incubation [57]. Several oleaginous yeast species, such as *Lipomyces starkeyi* and *Yarrowia lipolytica*, have been reported to produce lipase and their utilization as a catalyst for biodiesel (FAME) production has also been reported [73]. However, in the present study, the oleaginous yeast species *Meyerozyma caribbica*, isolated from wood-feeding termite symbionts, was identified for the first time as a cellulase, xylanase and lipase producer, suggesting that the MnP-producing oleaginous yeasts *Meyerozyma caribbica* could be potential candidate for valorizing lignocellulosic/fatty wastes into high added-value products.

### Tolerance to lignin degradation inhibitors

**Fig. 3:**
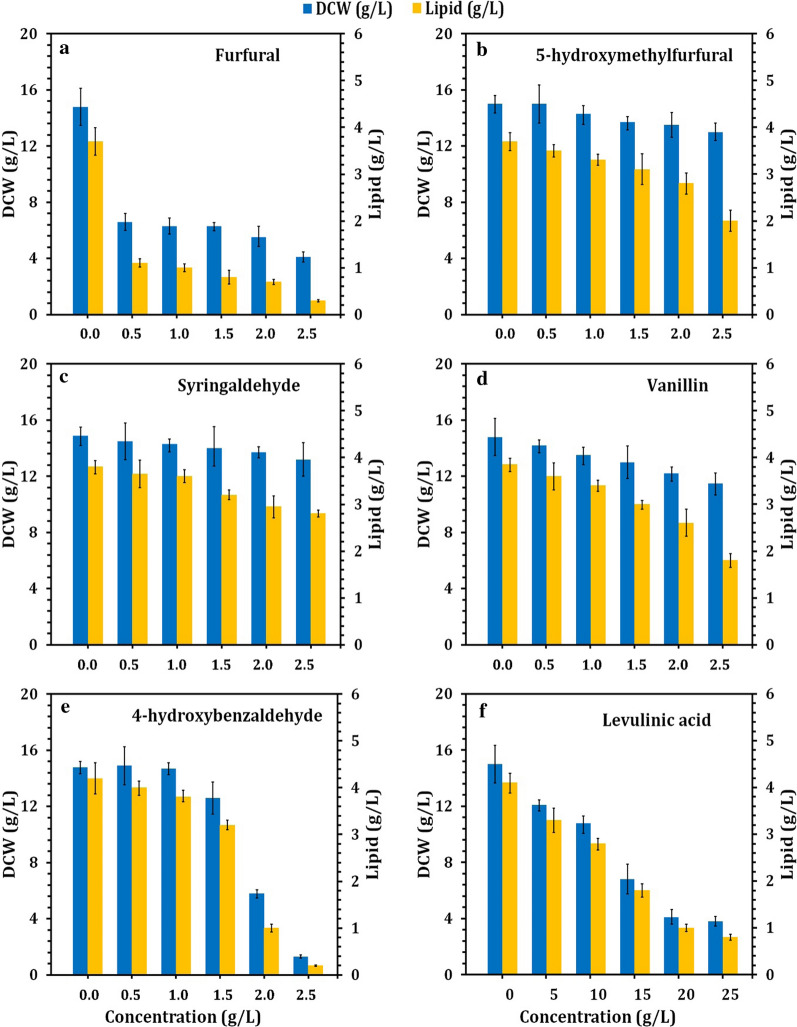
Tolerance of the MnP-producing oleaginous yeast *M. caribbica* strain SSA1654 to lignocellulose derived inhibitors simultaneously with lipid production. Furfural (**a**), 5-hydroxymethylfurfural (**b**), syringaldehyde (**c**), vanillin (**d**), 4-hydroxybenzaldehyde (**e**) and levulinic acid (**f**). Values are mean of three independent replicates and the standard deviation is indicated by error bars

Lignin is a heterogeneous biopolymer mainly consisting of syringyl, guaiacyl and hydroxyphenyl units [74]. Various microorganisms can bio-transform lignin and lignocellulose feedstocks into lipids, the raw material for biodiesel production [75–77]. However, various inhibitory compounds are generated, including weak acids (acetic acid, levulinic acid, and formic acid) and furans (furfural and 5-hydroxymethylfurfural), and phenolic compounds (syringaldehyde, 4-hydroxybenzaldehyde, and vanillin) [78–80]. The tolerance to lignin degradation inhibitors is considered as highly technical challenge for lipid production [81]. Therefore, screening of oleaginous yeasts using lignin or lignocellulose feedstocks is of great importance to accumulate high lipid content simultaneously with tolerance to various inhibitors. Oleaginous yeasts have been previously reported for their high tolerance to lignocellulosic degradation inhibitors [82–84]. This study provides the first insight into the tolerance of peroxidase-producing oleaginous yeast strains isolated from termite gut symbionts to various inhibitors for lipid production for future industrial applications. The tolerance comparison of the four MnP-producing oleaginous yeast strains *Meyerozyma caribbica* SSA1654, *Meyerozyma guilliermondii* SSA1547, *Debaryomyces hansenii* SSA1502, and *Vanrija humicola* SSA1514 to six inhibitory compounds (furfural, 5-hydroxymethylfurfural, vanillin, 4-hydroxybenzaldehyde, syringaldehyde, and levulinic acid) was evaluated. Between those oleaginous yeasts, *Meyerozyma caribbica* SSA1654 showed a high surviving potential in the presence of high lignin degradation inhibitors, while *Vanrija humicola* SSA1514 showed the lowest tolerance (data not shown). The performance of *Meyerozyma caribbica* SSA1654 to tolerate lignin inhibitors as well as to accumulate lipids is presented in Fig. 3.

Compared to the control, the dry cell weight (DCW) and lipid content of *Meyerozyma caribbica* SSA1654 were significantly decreased, since at a concentration of 0.5 g/L, furfural reduced the DCW and lipid content by 55.4 and 66.7%, respectively (Fig. 3a). However, the other furan inhibitor (5-hydroxymethylfurfural) showed less toxicity on *Meyerozyma caribbica* SSA1654 compared to furfural, in which DCW and lipid content were insignificantly decreased with increasing the concentration of 5-hydroxymethylfurfural, resulting in reduction of 13.3% (DCW) and 47.5% (lipid content) in the presence of 2.5 g/L of 5-hydroxymethylfurfural (Fig. 3b). It has been reported that furfural and 5-hydroxymethylfurfural are reduced to furfuryl alcohol and 5-hydroxymethylfurfural alcohol, respectively, and then oxidized back to furfural and 5-hydroxymethylfurfural but at very low concentrations, which are not harmful to the oleaginous yeast growth [80].

On the other hand, the performance of *Meyerozyma caribbica* SSA1654 to tolerate the three typical phenolic aldehydes, namely syringaldehyde, vanillin and 4-hydroxybenzaldehyde, was studied to represent the lignin derivatives of syringyl group (S), guaiacyl group (G) and hydroxyphenyl group (H), respectively (Fig. 3c–e). Syringaldehyde showed no significant difference with negligible inhibition on the DCW and lipid content of *Meyerozyma caribbica* SSA1654, up to a concentration of 2.5 g/L (Fig. 3c), while vanillin showed partial inhibition of the yeast growth and lipid content up to the concentration of 2.5 g/L (Fig. 3d). However, 4-hydroxybenzaldehyde significantly inhibited the DCW at 2.0 g/L, while both DCW and lipid content were almost completely inhibited at 2.5 g/L of 4-hydroxybenzaldehyde (Fig. 3e). Similar to furfural and 5-hydroxymethylfurfural, the three typical phenolic aldehydes (syringaldehyde, vanillin and 4-hydroxybenzaldehyde) are converted into their corresponding alcohols, then the acids are finally converted into acetyl-CoA before being assimilated into the tricarboxylic acid (TCA) cycle [80]. On the other hand, levulinic acid led to a significant reduction in the DCW and lipid content of *Meyerozyma caribbica* SSA1654 with increasing the concentration of levulinic acid up to 25 g/L (Fig. 3f). Weak acid inhibitors are converted into acetyl-CoA, then ultimately enter the TCA cycle [80]. Clearly, the robustness of *Meyerozyma caribbica* SSA1654 could be demonstrated by comparing it with other oleaginous yeast strains under the stress of lignin degradation inhibitors. Vanillin (2 g/L) and 4-hydroxybenzaldehyde (1.2 g/L) led to complete inhibition of *Rhodosporidium toruloides* Y4 and *Rhodosporidium toruloides* AS2.1389, while syringaldehyde partially inhibited the growth of both yeast strains (decreased by 15.7%) at a concentration of 2.19 g/L [85, 86].

### Performance of oleaginous yeast consortium NYC-1 for dye decolorization

Bioremediation of textile wastewater as a cheap source for water containing organic dyes (a potential carbon source) and successive biodiesel production can fulfill the energy requirements of textile dye industry [15, 87, 88]. Besides, biological conversion could serve as a potential platform for upgrading lignin or lignin-like dyes. In this scenario, application of microbial consortia and their enzymatic system is of great importance for utilizing various aromatics [76]. Therefore, a new oleaginous yeast consortium NYC-1 which stands for the molecularly identified yeast strains *Meyerozyma caribbica* SSA1654, *Meyerozyma guilliermondii* SSA1547, *Debaryomyces hansenii* SSA1502, and *Vanrija humicola* SSA1514 was successfully constructed in this study. As far as we know, many strains of *guilliermondii*, *humicola* and *hansenii* play an important role in degradation of azo dyes, such as *Meyerozyma guilliermondii* Y011, *Vanrija humicola* D206, and *Debaryomyces hansenii* F39A [15, 89, 90]. However, none of the previous studies has reported the biodegradation of textile azo dyes using constructed oleaginous microbial consortia. Interestingly, *Meyerozyma caribbica* has not been previously described in the literature to decolorize azo dyes. Therefore, the performance of the MnP-producing oleaginous yeast consortium NYC-1 to decolorize various industrial azo dyes viz. 10 azo dyes, i.e. Acid Orange 7 (AO7), Reactive Green 19 (RG19), Scarlet GR (SGR), Reactive Black 5 (RB5), Methyl Orange (MO), Reactive Blue 19 (RB19), Reactive Blue 81 (RB81), Methyl Red (MR), Reactive Red 120 (RR120), and Reactive Violet 5 (RV5) was studied and the results are depicted in Additional file 1: Table S1. The average decolorization rate of such azo dyes for the NYC-1 consortium was significantly higher than that observed for its pure cultures (data not shown). Enhanced rate of dye decolorization using bacterial and/or yeast consortia has been reported earlier [15, 18, 91, 92]. On the other hand, it has been observed that AO7 could be decolorized within 6 h with a maximum decolorization of 98.34%, while the consortium NYC-1 required more time (24 h) to achieve 83% decolorization of RB81 dye (Additional file 1: Table S1). To fully understand the mechanism, further study was carried out using AO7. Clearly, autoclaved cells of the constructed consortium NYC-1 and its individual yeast strains could not exhibit any decolorization of AO7, indicating the biological mechanisms of AO7 decolorization rather than  adsorption.

### Effect of initial dye concentration on decolorization performance

**Fig. 4:**
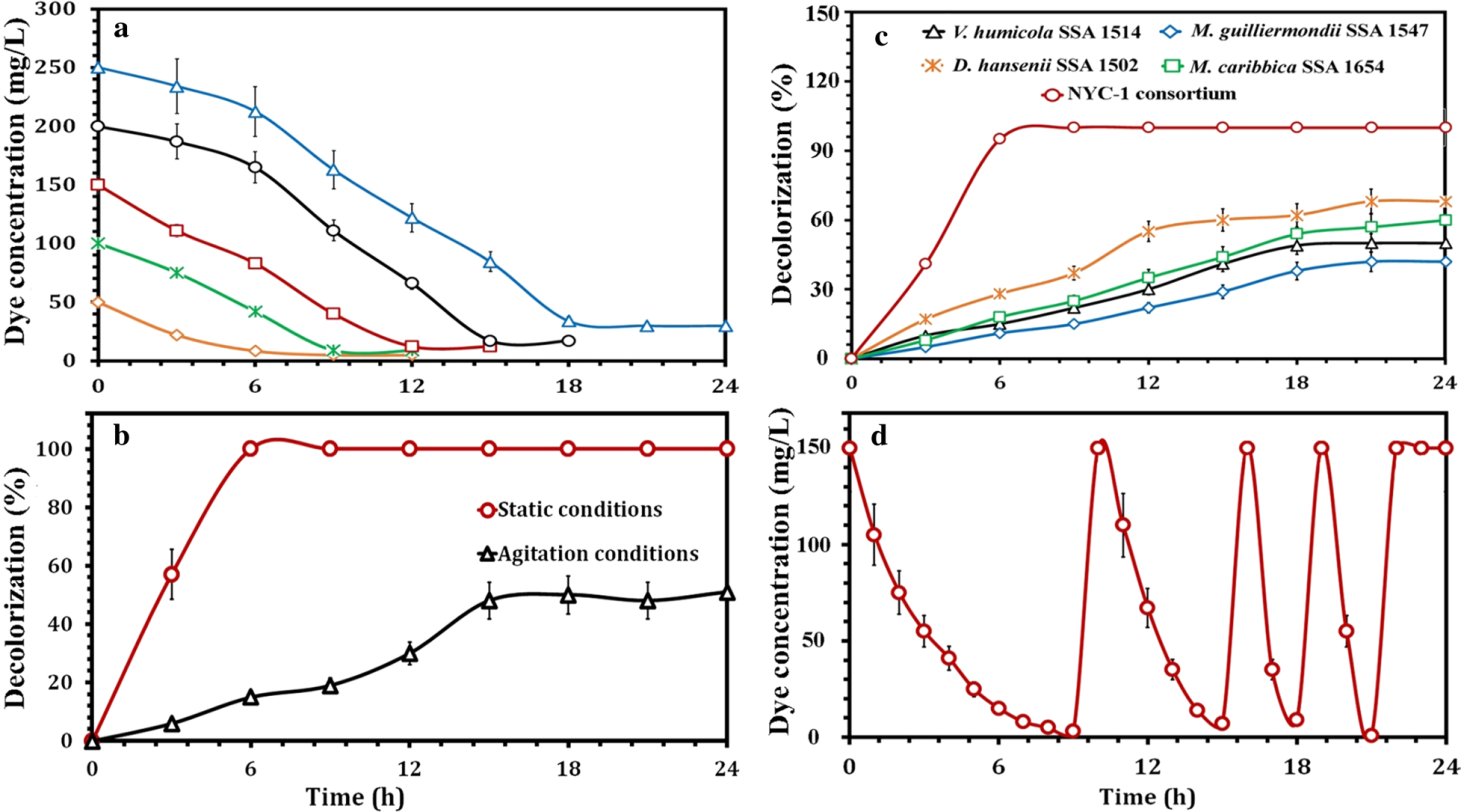
Performance of NYC-1 consortium to decolorize AO7 at different dye concentrations (**a**) and under static and agitation conditions (**b**). Decolorization performance of AO7 (at a concentration of 150 mg/L) by the NYC-1 consortium as compared to its individual strains (*M. caribbica* SSA1654, *M. guilliermondii* SSA1547, *D. hansenii* SSA1502, and *V. humicola* SSA1514) under static conditions (**c**). The repeated decolorization of AO7 (at a concentration of 150 mg/L) by NYC-1 consortium under static conditions (**d**)

The decolorization of dye AO7 was greatly influenced by dye concentration. More than 98% of AO7 decolorization was achieved by NYC-1 consortium within 3 h. However, the decolorization efficiency decreased with increasing AO7 concentration up to 250 mg/L, achieving a maximum percentage decolorization of more than 92% within 18 h (Fig. 4a). The efficiency of dye decolorization and dye concentration are inversely correlated [13, 15, 22, 93]. In total, the level of azo dyes (10–200 mg/L) in wastewater and aquatic environments is aesthetically unpleasant [14] and cause several biological issues, therefore the effective treatment of azo dyes is of utmost importance before being discharged into the environment. As the highest concentrations of azo dyes tested in this study exceed the dye levels in wastewater and aquatic environments, the newly constructed oleaginous yeast consortium NYC-1, could potentially be utilized for bioremediation of wastewaters with high concentration of azo dyes.

### Effect of static and agitation conditions on decolorization performance

The performance of NYC-1 consortium to decolorize AO7 under static and agitation conditions was tested and compared. Although oxygen has a significant effect on the physiological characteristics of the cells during their growth, the presence of oxygen can either favor or inhibit the microbial degradation of azo dyes [16, 94]. As depicted in Fig. 4b, the NYC-1 consortium decolorized AO7 efficiently (approximately complete decolorization) after 6 h of incubation under static conditions, whereas above 48% decolorization was achieved after 15 h of incubation under agitation conditions (120 rpm). This was probably due to the synergistic metabolic activities and production of enzymes by NYC-1 for the decolorization of AO7 dye under static conditions [95]. On the other hand, the growth of NYC-1 consortium under agitation conditions was fast as compared to its growth under static conditions, which was probably due to the facultative nature of yeast cells to grow anaerobically. Several studies reported efficient dye decolorization under static conditions when compared with agitation conditions [96–98]. The mechanism of azo dye biodegradation to its corresponding amines is initiated by the reduction of azo linkage using azoreductase [91]. Under agitation conditions, the excess of oxygen deprives azoreductase from receiving electrons required for the cleavage of azo linkage, while under static (microaerophilic) conditions, these electrons are readily available [99]. On the other hand, MnP is one of ligninolytic enzymes involved in dye decolorization processes. The MnP-producing fungal consortium successfully decolorized Cresol Red with 93% efficiency after 144 h of incubation [100].

Decolorization performance of 150 mg/L AO7 by the NYC-1 consortium as compared to its individual strains (*Meyerozyma caribbica* SSA1654, *Meyerozyma guilliermondii* SSA1547, *Debaryomyces hansenii* SSA1502, and *Vanrija humicola* SSA1514) under static conditions was also evaluated. Clearly, SSA1654, SSA1547, SSA1502, and SSA1514 could decolorized the model dye tested. However, the decolorization of AO7 by NYC-1 was significantly higher than that of all individual strains (Fig. 4c), which was probably due to the concerted metabolic activities of the individual strains constituting NYC-1 consortium [15, 16, 101]. Furthermore, repeated spread plate studies revealed that no dominant yeast strain was observed over a period of nine months, suggesting the cooperation and synergistic metabolic activities among individual strains constituting NYC-1 consortium.

### Effect of repeated cycles of dye addition on decolorization performance

The repeated decolorization of AO7 (at a concentration of 150 mg/L) under static conditions was also studied. It has been observed that no significant variation in the decolorization of AO7 by NYC-1 consortium was observed after the fourth addition (Fig. 4d). The microorganism seems to initially use nutrient broth medium (as a carbon source), resulting in a slow decolorization in the first cycle of addition. With increase in number of microbial cells and less availability of nutrients in the culture medium, the microorganism seems to utilize the dye itself (as a carbon source), then gradually acclimatize to this new source, resulting in faster decolorization in the subsequent cycles of dye addition [102]. On the other hand, after the first cycle of dye addition, the formation of some metabolites as well as reduction in pH caused by the microorganism seems to be not only non-toxic to the microorganism, rather facilitating in dye biodegradation. Therefore, the microorganism once triggered for decolorization can use the dye as carbon source [102, 103]. After the fourth cycle of dye and fresh medium additions, color removal was recovered, suggesting that the cell biomass recovered its full enzymatic activity and could be thus reused for multiple decolorization cycles and subsequently for large scale bioremediation purposes [18, 97].

### Effect of heavy metals on decolorization performance

The performance of NYC-1 consortium to decolorize AO7 in the presence of several heavy metals (MnSO_4_, CuSO_4_ and FeSO_4_; 100 mg/L each) was also studied. NYC-1 showed maximum decolorization of 98.76% with Mn after 24 h of incubation, which was probably due to the induction of Mn to MnP activity of NYC-1 consortium, resulting in the enhancement of the degradation of chemical nature of azo dye [104]. On the other hand, NYC-1 consortium revealed maximum decolorization of 87.34 and 65.18% with Cu and Fe, respectively. In the presence of Fe at higher concentration, enzymatic activities may become unstable, hence affecting dye decolorization efficiency [105]. Our findings are in accordance with those previously reported by Singh and Dwivedi [22].

### Effect of supplementation of carbon and nitrogen sources on decolorization performance

Carbon and nitrogen sources have an important influence on the extent of dye biodegradation. Since dyes are deficient in carbon, it seems necessary to supplement extra carbon or nitrogen source to assist biodegradation of azo dyes [106]. Therefore, in this study, the decolorization performance of AO7 by the yeast consortium NYC-1 was studied in the presence of different carbon sources (glucose, xylose, sucrose, maltose, and starch) and nitrogen sources (yeast extract, beef extract, peptone, urea, and sodium nitrate) to obtain efficient and faster dye decolorization. While trying to enhance decolorization performance of AO7 by NYC-1 consortium, extra carbon sources were supplied in the Bushnell Hass (BH) synthetic medium [18]. Clearly, the NYC-1 consortium showed lower decolorization performance (approximately 17%) within 24 h in the BH medium since no co-substrate was supplemented to this synthetic medium. Among different carbon sources tested for efficient decolorization of AO7, xylose and glucose were found to be better carbon sources with maximum decolorization percentage of 98.25 and 94.18%, respectively. Monosaccharides, such as glucose and xylose, are easily available and effective carbon sources for microbial metabolism. Yang et al. [107] found that the addition of glucose improved the efficacy of azo dye degradation. However, addition of maltose (77.34%), sucrose (69.71%) and starch (53.41%) found to be less effective to promote the decolorization performance of the NYC-1 consortium as compared to the monosaccharides tested, which was probably due to the preference of the constructed yeast consortium in assimilating the added di- and polysaccharides over using AO7 dye as a carbon source [98, 108]. Carbon sources provide energy for the growth and survival of the microorganisms together with their importance as electron donors, which are necessary for the breakage of the azo linkage [109]. During the decolorization process, the generated reducing equivalents, such as Flavin nucleotide (FAD), are transferred to the dye. Such reducing equivalents work as an electron shuttle between the dye and the NADH‑dependent azo reductase during the electron transport chain of the microbial metabolism [110]. The decolorization performance of AO7 by the yeast consortium NYC-1 in the presence of nitrogen sources was also studied. The maximum decolorization percentage was observed in the presence of yeast extract (100%), and peptone (97.15%), within 24 h. Yeast extract and peptone were reported to trigger the expression of NADH, which acts as an electron donor for the reduction of azo dyes, and thus decolorize the dye effectively [111]. The addition of urea and sodium nitrate to the BH medium strongly inhibited AO7 decolorization by 44.32 and 38.80%, respectively, while no decolorization was observed in the presence of ammonium chloride within 24 h, which was probably due to their inhibitory effect on the enzyme systems involved in the dye decolorization process [112].

### Effect of co-substrate supplementation on decolorization performance

The search for cheaper supplementary sources, such as agricultural wastes, would be essential to enhance process efficiency. In this regard, the decolorization performance of AO7 in the presence of extracts of agro-wastes was also evaluated. The highest decolorization performance by the MnP-producing yeast consortium NYC-1 was observed with rice straw (92.33%), sorghum husk (86.17%), wheat bran (72.59%), and rice stalk (59.83%). Saratale et al. [113] and Jadhav et al. [114] reported that the presence of lignocellulosic substrates enhances decolorization through effective production of ligninolytic enzymes. Similar findings were reported by Saratale et al. [115] who found that supplementation with rice husk and rice straw extract enhanced the decolorization performance of the developed bacterial consortium GR on Scarlet R. The significant dye bio-decolorization in the presence of agricultural residues (e.g. wheat bran) in basal medium was probably due to the presence of certain components in wheat bran, which act as electron donors for the decolorization of azo dyes [116]. In total, supplementation of the cultivation medium with agro-wastes could enhance decolorization of AO7 by the NYC-1 consortium, along with producing ligninolytic enzymes. On the other hand, the addition of bagasse extract to the BH medium strongly inhibited AO7 decolorization by 28.62%, which was probably due to the production of alcohols or volatile organic acids as electron donors for reducing the dye. Lastly, no decolorization was observed in the presence of soybean husk and corn stalk, which was probably due to their inhibitory effects on microbial enzyme systems involved in dye decolorization. Therefore, it could be concluded that the use of lignocellulosic agro-wastes (rice straw, sorghum husk and wheat bran) instead of pure co-substrates (yeast extract, peptone, xylose, glucose and maltose) for the enhancement of AO7 decolorization represents an inexpensive and eco-friendly process, concurrently providing a solution to the challenge of disposal of agro-wastes that are present in huge quantities worldwide [98].

### Effect of dye on the fatty acid profile of NYC-1 consortium

Bioremediation of textile wastewater is an emerging technology, which uses microorganisms (e.g. bacteria, filamentous fungi, yeasts, and algae) for the treatment of dyes. Among these microorganisms, oleaginous yeasts have received increasing attention for efficient degradation of textile wastewater and successive accumulation of fatty acids which can be extracted and used for biodiesel production. Therefore, this study might be the first to explore the efficacy of a MnP-producing oleaginous yeast consortium for coupling dye biodegradation and biodiesel production. The extracted lipids of the AO7-degraded NYC-1 consortium were compared with that of the control (NYC-1 consortium). Clearly, a decrease in the saturated fatty acid especially dodecanoic acid percentage as well as increased amount of other alkenes and alkanes in the dye-treated oleaginous yeast consortium compared with the control one were observed (Additional file 1: Table S2). On the other hand, the main physicochemical properties of biodiesel produced by AO7-degraded NYC-1 consortium were estimated and compared with the ASTM biodiesel standard (US) and diesel standard (Additional file 1: Table S3). In this study, the biodiesel extracted from NYC-1 consortium showed a better density as compared with that of jatropha [117], the marine cyanobacteria *Synechococcus* sp. [118], and *Geitlerinema* sp. TRV27 [119]. In addition, kinematic viscosity of the biodiesel extracted from NYC-1 consortium (4.38 mm^2^/s) was quite similar to that of *Geitlerinema* sp. TRV27 and *Synechococcus* sp., which were found to be 3.36 and 3.13 mm^2^/s, respectively [119, 120]. The biodiesel extracted from NYC-1 consortium showed less acid value (0.273 mg of NaOH/g of oil) as compared to jatropha oil diesel (5.31 mg of NaOH/g of oil) [121], and sorghum oil diesel (0.434 mg of NaOH/g of oil) [120]. As shown in Additional file 1: Table S3, cetane value of the biodiesel extracted from NYC-1 consortium was found to be 53 as compared to that of palm (62), sunflower (49), rapeseed (54) and peanut (54) [122]. Clearly, the physicochemical properties of the biodiesel produced by the dye-treated NYC-1 were found to be within the standard limits.

### MnP and Lac activities under different concentration of AO7 azo dye

Except azoreductase, ligninase enzymes MnP and Lac are effective agents for dye biodegradation, generating more eco-friendly products [123]. Several Lac-producing fungal isolates, which degraded various dyes of different chemical structures, have effectively catalyzed the phenolic substrate oxidation of dye compound using oxygen as electron acceptor [124]. The application of such biological agents promotes an eco-friendly technology as well as the reduction in chemical load [125]. As depicted in Table 5, the activity of Lac enzyme increased with increasing the concentration of AO7, which ranged from 0.69 ± 0.16 to 1.53 ± 0.12 µm/L after 120 h of dye-degrading NYC-1 consortium, suggesting that Lac enzyme could be effectively involved in the biodegradation of AO7 by the oleaginous consortium developed in this study. Pandey et al. [126] reported the effectiveness of various oxidoreductase enzymes in the decolorization process, converting parent dyes into less toxic compounds. Similar to Lac, MnP activity also increased with increasing the concentration of AO7, which ranged from 1.66 ± 0.011 to 4.56 ± 0.02 µm/L after 120 h of dye-degrading NYC-1 consortium, hence inducing the degradation of AO7 (Table 5). Gao et al. [127] reported that MnP related to lignolytic peroxidase family is able to mineralize azo dyes via redox reaction, which oxidizes phenolic compounds with conversion of Mn^+2^ to Mn^+3^. On the other hand, H_2_O_2_ content increased with increasing the concentration of AO7, reaching 6.0 ± 0.07 nM/g FW at 500 mg/L dye concentration, then decreased, achieving 2.0 ± 0.03 nM/g FW at a AO7 concentration of 1000 mg/L after 120 h of dye-degrading NYC-1 consortium. It has been reported that increased amount of H_2_O_2_ as cofactors is required for activating peroxidase enzyme but at a certain time, resulting in dye biodegradation by MnP, then any further increase can inhibit the enzyme activity [128].

**Table 5:** Ligninase activities and H_2_O_2_ content under different concentrations of dye-treated consortium NYC-1

AO7 concentration (mg/L)	MnP activity (µm/L)	Laccase (µm/L)	H_2_O_2_ content (nM/g FW)
0.0	1.66 ± 0.011^a,b^	0.69 ± 0.16	0.91 ± 0.07
100	2.48 ± 0.015	0.40 ± 0.11	4.0 ± 0.05^a,c^
500	3.77 ± 0.011	0.96 ± 0.14	6.0 ± 0.07^a,c^
1000	4.56 ± 0.020^a,b^	1.53 ± 0.12	2.0 ± 0.03

The values are mean of three independent experiments; ^a^Significant difference versus laccase; ^b^Significant difference versus H_2_O_2_ content; ^c^Significant difference versus MnP activity

### Possible dye degradation pathway coupled with biodiesel production

**Fig. 5:**
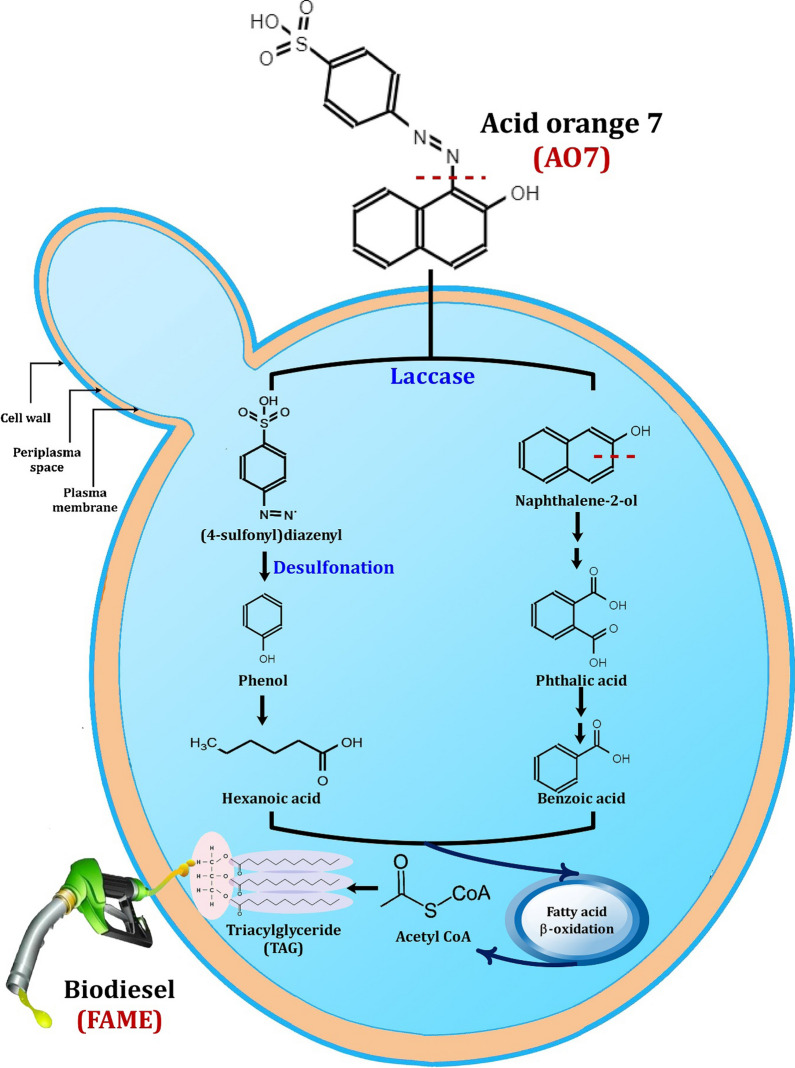
The possible pathway of azo dye (AO7) degradation by the oleaginous yeast consortium NYC-1 linked with biodiesel production

The mechanism of azo dye biodegradation and successive production of biodiesel was proposed based on GC–MS technique, ligninase activities and previous studies (Fig. 5). According to our proposal, the first step in the degradation mechanism of AO7 by the developed oleaginous yeast consortium NYC-1 is the decolorization of AO7 by asymmetric cleavage of the azo bond (–N=N–) as catalyzed by Lac, producing two intermediates namely (4-sulfophenyl)diazenyl and naphthalen-2-ol (Fig. 5). The first intermediate is subjected to oxidative desulfonation, resulting in the formation of phenyl diazene, which is an unstable radical and it might rapidly lose nitrogen as gas molecule. Hence, phenol is formed as a result of the nucleophilic substitution of hydroxyl radical on the aromatic ring. The formation of hexanoic acid (R_*t*_, 5.08; M + H^+^, 60) might be due to the oxidative ring cleavage of phenols, which are the natural substrates for Lac. Hexanoic acid is a carboxylic acid with the general formula C_5_H_11_COOH. It is a non-toxic fatty acid found naturally in many food products available for human consumption, various animal fats and oils. On the other hand, the second intermediate (naphthalen-2-ol) is subjected to aromatic ring cleavage, producing phthalic acid (R_*t*_, 11.02; M + H^+^, 149), which is further oxidized to form benzoic acid (R_*t*_, 7.45; M + H^+^, 105) as depicted in Fig. 5. Zhang et al. [129] reported that the addition of *p*-methyl benzoic acid was able to increase the growth of oleaginous yeasts, resulting in higher lipid accumulation. The intermediate products (4-sulfophenyl) diazenyl and naphthalen-2-ol may directly or indirectly enter fatty acid β-oxidation reactions to produce NADH_2_ and FADH_2_, which may be used in ATP synthesis. Besides, acetyl CoA is a precursor necessary for TAG synthesis and the accumulated TAG can be effectively transesterified to further produce biodiesel. Similar pathway was also observed for the decolorization of synthetic dyes, such as Remazol brilliant blue, by *Pleurotus ostreatus* HAUCC 162 Lac [130]. The formation of phenyl diazene radical was also observed as a result of biodegradation of phenolic azo dyes based on fungal Lac [131]. AO7 oxidation by Lac of the NYC-1 consortium was subjected to reactive free radicals as illustrated above (Fig. 5). Besides, MnP catalyzes a H_2_O_2_-dependent oxidation of Mn^2+^ to form highly reactive Mn^3+^, which subsequently oxidizes the phenolic parts of lignin to produce free radicals [132]. Therefore, the mechanism proposed for the degradation of AO7 azo dye by the NYC-1 consortium with successive biodiesel production revealed the formation of phenol-based products thereby avoiding the formation of toxic aromatic amines (Fig. 5).

## Conclusion

Wood-feeding termite symbionts represent a significantly rich source of yeasts which can be isolated and utilized for various applications, including wastewater remediation and production of energy and added-value products. Owing to yeasts’ multiple valuable properties, such as high enzymatic activity, tolerance to lignin-derived metabolites and lipid accumulation, a combined process including azo dye degradation and biodiesel production is proposed, in the framework of the present study. First, 38 strains were isolated and screened for MnP production, along with lipid accumulation. The highest lipid content determined was 47.25% in case of *Meyerozyma caribbica*, while the produced biodiesel was in compliance with international standards. Furthermore, *Meyerozyma caribbica*, *Meyerozyma guilliermondii*, *Debaryomyces hansenii*, and *Vanrija humicola* were identified for the first time as cellulase, xylanase, and lipase producers. Subsequently, the constructed consortium NYC-1 was evaluated for azo dyes remediation, while static conditions, addition of Mn, supplementation with xylose and glucose as carbon sources, yeast extract and peptone as nitrogen sources, or rice straw as an agro-waste co-substrate enhanced decolorization activity. Last, a dye degradation pathway coupled to biodiesel production was proposed, which included the formation of phenol-based products instead of toxic metabolites. In total, this study might be the first to explore the application of a MnP and lipid-producing consortium for coupling dye degradation and biodiesel production.

## Methods

### Dyestuff, chemicals and agricultural wastes

The chemicals, reagents, and azo dyes used in this study were purchased from Sigma-Aldrich (St. Louis, MO, USA). All chemicals and reagents were of analytical grade. The azo dyes tested in this study were AO7 (*λ*_max_ = 484 nm), RB5 (*λ*_max_ = 595 nm), RB19 (*λ*_max_ = 592 nm), RR120 (*λ*_max_ = 537 nm), RG19 (*λ*_max_ = 630 nm), MO (*λ*_max_ = 465 nm), MR (*λ*_max_ = 424 nm), SGR (*λ*_max_ = 511 nm), RV5 (*λ*_max_ = 530 nm), and RB81 (*λ*_max_ = 581 nm). To optimize culture conditions for the enhancement of AO7 decolorization, different carbon sources (glucose, xylose, sucrose, maltose, and starch) and nitrogen sources (yeast extract, beef extract, peptone, urea, and sodium nitrate) were used. Different agricultural wastes (sorghum husk, soybean husk, corn stalk, rice stalk, rice straw, bagasse, and wheat bran) obtained from local farmers and industries (Zhenjiang, China) were also used in this study to evaluate their effects on the dye decolorization of AO7.

### Screening and enrichment process

**Fig. 6:**
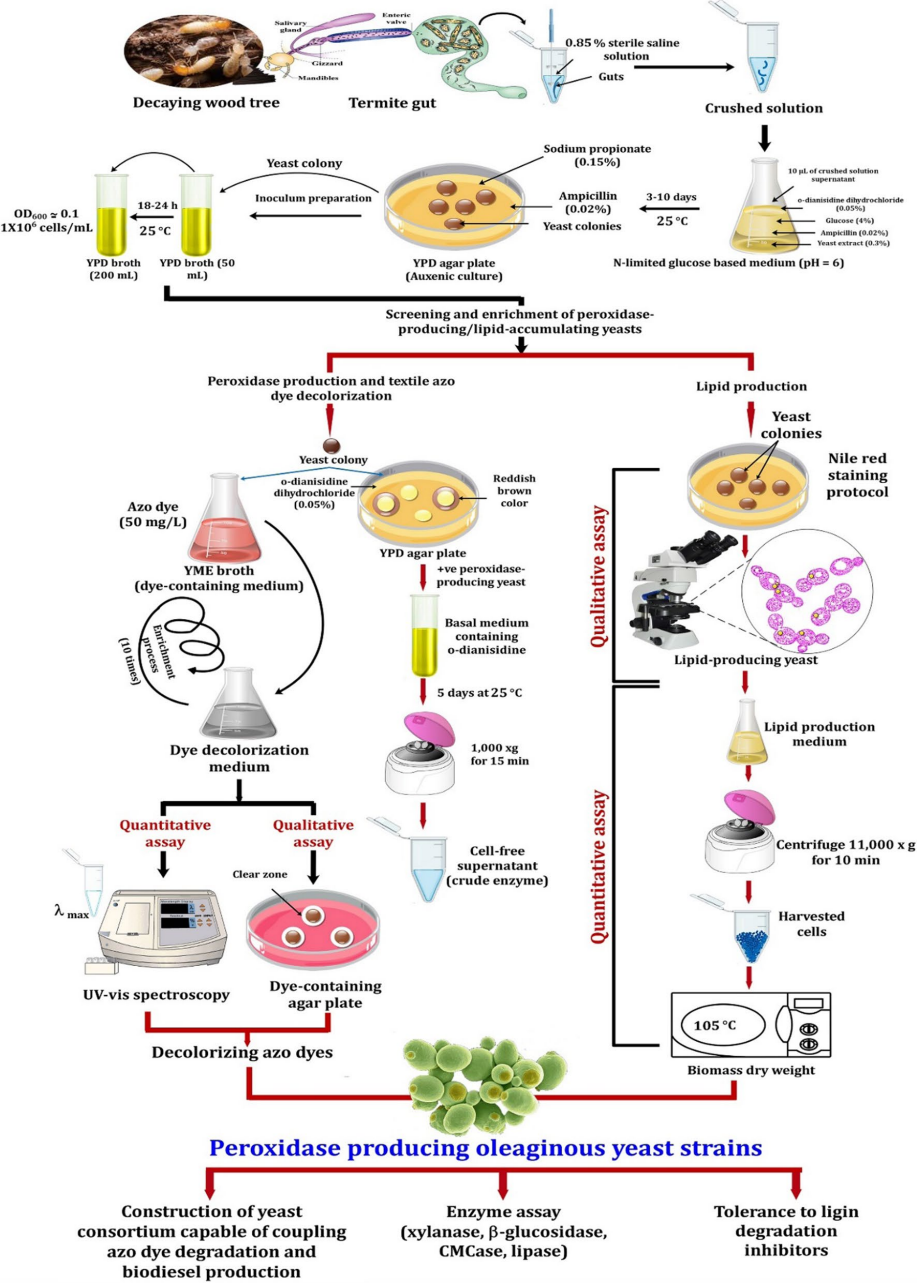
Experimental set up for screening and enrichment of MnP-producing oleaginous yeasts valued for textile azo dyes decolorization, lignin valorization and biodiesel production

The wood-feeding termite, *R. chinenesis* was collected from rotting wood trees (Wuhan, Hubei, China), while *C. formosanus* termite samples were collected from three different sites at Suzhou, Nanjing and Zhenjiang (China). Isolation of yeasts from insect gut symbionts was performed following Suh and Blackwell [133]. Briefly, the surface of termite species was sterilized with 70% ethanol for 1 min before dissection. Then, guts of the collected termite samples were removed aseptically, following with homogenization using saline solution (0.85%, w/v NaCl). To screen and characterize stable peroxidase-producing yeasts capable of coupling azo dye biodegradation and biodiesel production, an enrichment process was performed (Fig. 6). Since a high carbon to nitrogen (C/N) ratio improves lipid accumulation, N-limited medium was used following the protocol of Suutari et al. [134]. Briefly, isolation of yeasts was carried out in a conical flask (100 mL) containing 40 mL of N-limited glucose-based medium (6.8 g/L KH_2_PO_4_, 2.45 g/L NaH_2_PO_4_, 1.72 g/L MgSO_4_·7H_2_O, 0.067 g/L MnSO_4_·7H_2_O, and 0.2 g/L CaCl_2_·2H_2_O) supplemented with 0.5 g/L o-dianisidine dihydrochloride (a precursor of many azo dyes). Glucose (C-source) was added to the medium at a final concentration of 40 g/L, while yeast extract (3.0 g/L) was used as the only N-source, yielding a C/N ratio of around 40. A volume of 10 µL of the crushed gut solutions was inoculated in the prepared flasks containing N-limited glucose-based medium. Then, flasks were incubated at 25 °C for 3–10 days with an agitation speed of 150 rpm. A total of 75 yeast colonies were isolated on YPD medium (10 g/L yeast extract, 20 g/L peptone, 10 g/L dextrose and 20 g/L agar) containing 0.2 g/L ampicillin (antibacterial agent) and 1.5 g/L sodium propionate (antifungal agent).

### Isolates’ identification and phylogenetic analysis

Yeast isolates were identified by amplified rDNA gene sequencing following the procedures described earlier [39]. The isolated DNA was amplified using NL1/NL4 and ITS1/ITS4 primers, for D1/D2 and ITS regions, respectively. PCR amplification was performed as previously described [91]. Phylogenetic and evolutionary analyses were conducted in MEGA software version 7.0. The identification of yeast strains was performed based on nucleotide BLAST (http://www.ncbi.nlm.nih.gov/BLAST/) database.

### Peroxidase production

Qualitative assay of peroxidase-production was determined on YPD agar plates supplemented with 0.5 g/L o-dianisidine dihydrochloride and incubated at 25 °C, up to 3 days. The reddish-brown color change around yeast colonies, after flooding the plates with 0.4 mM H_2_O_2_, is positively correlated with the production of peroxidase enzyme. Subsequently, the peroxidase-producing yeast isolates, which showed a large reddish-brown color, were selected for enzyme production in basal medium contained (per liter): 1.0 g o-dianisidine, 1.5 g yeast extract, 15 g dextrose, 0.5 g KH_2_PO_4_, 0.5 g K_2_HPO_4_ and 0.5 g NaCl. In addition, MnSO_4_·4H_2_O (150 µM) was added to the basal medium to stimulate the production of MnP. The cultures were incubated at 25 °C for 5 days, followed by centrifugation at 10,000×*g* for 15 min to obtain crude enzyme (Fig. 6).

### Determination of glucose residual, nitrogen assimilation and lipid production

The isolated yeast colonies were qualitatively screened for lipid production by flooding the agar plates with 0.3% Sudan black B. After 30 min, excess stain was rinsed with 70% ethanol. The putative oleaginous yeasts which retained the blue color were further confirmed for lipid accumulation using Sudan black B and Nile red staining protocols (Fig. 6). The qualitative assay for lipid production with Sudan black B was performed according to Sagia et al. [84] and the intracellular lipid droplets were stained blue when observed under light microscope. On the other hand, lipid detection by Nile red fluorimetry was achieved following the protocol described by Vyas and Chhabra [57]. Lipid-producing yeast cells were observed under a fluorescence microscope (Olympus BX35) with emission wavelength of 460–500 nm.

Yeast isolates that were positive for lipid production were further quantitatively assessed through gravimetric analysis [55]. After 5 days of growth in lipid production medium at 25 °C, biomass was harvested using a centrifuge (model 5418, Eppendorf, Germany) at 11.000×*g* for 10 min and subsequently the pelleted biomass was washed three times with 10 mL of distilled water and dried at 105 °C until constant weight (Fig. 6). To quantify lipids in cell biomass at a dry weight basis, the sonicated cells were homogenized with 20 mL solvent mixture of chloroform and methanol (2:1 v/v). After 30 min of incubation at room temperature, the upper phase was discarded, while the lower chloroform phase containing the lipids was mixed with 10 mL of solvent mixture into a pre-weighted container. Subsequently, the solvent was evaporated using a rotary evaporator, dried using a rotary vacuum pump (model SHZ-D-III) for 10 min, cooled in a desiccator for 30 min, then the container was weighted again and lipid yield (g lipid/g biomass) was determined [135]. The parameters of biomass and lipid production were calculated using the following equations:

$$\mathrm{Biomass\,productivity}=\frac{\mathrm{Cell\,dry\,mas}}{\mathrm{ Culture\,volume}}(\mathrm{g}/\mathrm{L})$$

$$\mathrm{Lipid\,productivity }= \frac{\mathrm{Lipid\,output}}{\mathrm{ Days\,required\,to\,achieve\,maximum\,lipid}} (\mathrm{g}/\mathrm{L}/\mathrm{day})$$

$$\mathrm{Lipid\,output }=\frac{\mathrm{ Mass\,of\,lipid}}{\mathrm{Culture\,volume}}(\mathrm{g}/\mathrm{L})$$

$$\mathrm{Lipid\,content}=\frac{\mathrm{Lipid\,output}}{\mathrm{ Biomass\,productivity}}\times 100 (\mathrm{\%})$$

$${Y}_{\mathrm{L}/\mathrm{x}}=\frac{\mathrm{Lipid\,output}}{\mathrm{ Total\,biomass\,produced}} (\mathrm{g}/\mathrm{g})$$

$${Y}_{\mathrm{X}/\mathrm{S}} =\frac{\mathrm{Total\,biomass\,produces}}{\mathrm{ Consumed\,substrate}} (\mathrm{g}/\mathrm{g})$$

$${Y}_{\mathrm{L}/\mathrm{S}}=\frac{\mathrm{Cellular\,lipids\,produced}}{\mathrm{ Consumed\,substrate}} (\mathrm{g}/\mathrm{g})$$

Furthermore, the supernatant derived after cell harvesting was used for measuring the content of glucose and nitrogen throughout culturing time. Quantitative analysis of reducing sugars, such as glucose, was performed using the 3,5-Dinitrosalicylic acid method [136]. The concentration of ammonia in the culture supernatant was estimated to determine nitrogen assimilation following the method described by Gomez-Alonso et al. [137]. Briefly, derivatization was performed in the supernatant followed by ultra-performance liquid chromatography (UPLC, Waters, Milford, USA) equipped with the analytical column (Acquity BEH C18, Waters, Milford, USA). Before injection, samples were filtered using nylon filters (0.2 µm pore size).

### Characterization of extracted lipids and biodiesel properties

The extracted neutral lipids were analyzed by TLC using a K6 silica gel plate (Merck, India). The mixture of hexane: diethyl-ether: methanol: acetic acid (78:17:3:2, v/v) was used as solvent system for analyzing TAG. Olive oil was used as positive control of vegetable TAG for plate migration. TLC plates were then exposed to iodine vapor to detect lipids [138]. The extracted lipid was also subjected to methanolysis following the protocol described by Morrison and Smith (1964). The FAME obtained by transesterification was further analyzed on a gas chromatography system (Agilent Technologies, France, M-6890) equipped with a flame ionization detector FID. An aliquot (2 µL) was injected employing a HP-5 capillary column (30 m, 0.32 mm, 0.25 µm). Helium was used as carrier gas with a flow rate of 1 mL/min. Detector temperature was set at 290 °C, while the temperature of injector was 270 °C. The column temperature was set at 50 °C, increased to 180 °C at a rate of 13 °C/min and held for 3 min, then the temperature was increased to 280 °C at a rate of 10 °C/min and held for 4 min. The retention times of the produced FAME were compared with those of known standards (Sigma-Aldrich, St. Louis, MO, USA). The fatty acids composition of each sample was determined in triplicate. Also, the quality of the biodiesel was determined in this study. For this purpose, the main physicochemical properties were studied, including CN, IV, SV, *ν*, *ρ*, OS, LCSF and DU [61].

### Development of yeast consortium NYC-1

To construct a stable MnP-producing oleaginous yeast consortium capable of decolorizing textile azo dyes and successive production of biodiesel, the enrichment culture technique was performed as previously described by Al-Tohamy et al. [13] with minor modifications. Briefly, the MnP-producing yeast isolates that were positive for lipid production were inoculated into 100 mL Erlenmeyer flasks containing 30 mL YME medium (3.0 g/L yeast extract, 3.0 g/L malt extract, 5.0 g/L peptone, and 10 g/L dextrose) amended with different azo dyes at an initial concentration of 50 mg/L and incubated at 25 °C under static conditions. Once decolorization was observed, an aliquot (5 mL) was re-inoculated into a fresh dye-containing medium for additional round of culturing transfer. For 10 generations of successive enrichment process, a stable yeast consortium with high decolorization performance was obtained (Fig. 6). The dye decolorization procedures were also repeated on agar plates supplemented with 50 mg/L dye and incubated at 25 °C for 48 h. The fastest-growing yeast that was capable of decolorizing various azo dyes as well as showing a high ratio of decolorization zone to the yeast colony diameter was isolated for phenotypic and genotypic characterization [39], followed by consortium construction [101]. For constructing a novel yeast consortium, designated as NYC-1, 100 µL of overnight grown culture of each individual yeast strain was inoculated separately into 100 mL Erlenmeyer flasks containing 30 mL YPD broth and incubated at 25 °C under static conditions for 24 h. The individual yeast strains were then mixed in equal proportion (500 μL; OD of 0.2) to maintain the same cell count in the yeast consortium NYC-1 as well as in the pure culture.

### Enzyme assays

MnP activity was measured as previously described [139] using guaiacol as mediator. The obtained crude enzyme extract (600 µL) was mixed with 500 µL of 0.1 M guaiacol, 500 µL of sodium tartrate buffer (100 mM, pH 5), 50 µL of H_2_O_2_, and 500 µL of MnSO4, and then incubated at 30 °C for 5 min. The appearance of brown color after the addition of guaiacol is the indicator for the presence of peroxidase enzymes. The absorbance at 465 nm was monitored using a UV–vis spectrophotometer (Shimadzu UV2600, Japan). Lac activity was determined following the method described by Adnan et al. [140], using guaiacol as the corresponding substrate. The reaction mixture containing 500 μL of guaiacol solution was mixed with 1500 μL of sodium acetate buffer (10 mM, pH 5) and 500 μL of filtered crude enzyme extract, and then incubated at 30 °C for 3 min. The absorbance at 450 nm was monitored using a UV–vis spectrophotometer (Shimadzu UV2600, Japan). H_2_O_2_ content was determined as previously described [141]. Endo-β-1,4-glucanase (CMCase) and xylanase activities were determined by the 3,5-Dinitrosalicylic acid method [136] using CMC (1% w/v) and xylan, respectively [39]. For the enzymatic activity of β-glucosidase, *p*-nitrophenyl glucopyranoside (*p*-NPG) was used as the corresponding substrate (5 mM, pH 5) according to the method of Ali et al. [16]. Lipase activity was measured using *p*-nitrophenyl palmitate (*p*-NPP) as the corresponding substrate [18]. One unit of enzyme activity was defined as the amount of enzyme equivalent to the release of 1.0 μmol of the reaction product per minute, under the assay conditions.

### Tolerance to lignin degradation inhibitors

The peroxidase-producing oleaginous yeast strains were tested for their tolerance to common inhibitors in lignocellulosic hydrolysates. Each yeast strain was inoculated in Erlenmeyer flasks with sterile yeast nitrogen base broth [142], containing 1% glucose and supplemented with different concentrations of furfural, HMF, syringaldehyde, vanillin, and 4-hydroxybenzaldehyde. The flasks were incubated at 25 °C and pH 5 for 5 days. The tolerance of yeast strains to the inhibitors tested was measured at 600 nm and it was expressed as DCW (g/L). Percentage decrease in yeast growth due to the inhibitors was calculated compared to growth without inhibitors, used as control.

### Azo dye decolorization experiments

Quantitative assay of dye decolorization was performed following the method described previously [14], with a minor modification. Briefly, the respective *λ*_max_ of the different azo dyes was measured individually with a UV–vis spectrophotometer (Shimadzu UV2600, Japan). Un-inoculated control was included during the decolorization experiments. All experiments were carried out in triplicate. The above decolorization protocol was also followed while evaluating the efficacy of the NYC-1 consortium in the decolorization of AO7 at increasing initial dye concentrations (50–2000 mg/L), static and agitation conditions, repeated cycles of dye addition, and in the presence of heavy metals (MnSO_4_, CuSO_4_ and FeSO_4_). Dye decolorization performance was also assessed in the presence of different carbon sources (glucose, xylose, sucrose, maltose, and starch) and nitrogen sources (yeast extract, beef extract, peptone, urea, and sodium nitrate) with the addition of 0.5% (w/v) of each source to the culture medium. To study the effect of agricultural waste as co-substrates supplementation on the decolorization of AO7, different agricultural wastes (sorghum husk, soybean husk, corn stalk, rice stalk, rice straw, bagasse, and wheat bran) were used in this study (0.5 mL extract of 0.5% boiled agricultural residue). The effect of AO7 on the fatty acid profile of NYC-1 consortium was assessed by comparing the chemical profile of dye-decolorizing biomass with the control. After 120 h of incubation, the dye-decolorizing biomass was extracted using hexane solvent solution, and then transesterified by alkali catalyst method for lipid analysis.

### Identification of dye degradation metabolites

The products of AO7 degradation by NYC-1consortium were analyzed by gas chromatography/mass spectrometry (GC–MS). The samples were extracted with dichloromethane (DCM) using liquid to liquid extraction at various pH values to neutralize any charged compounds, such as phenolics and amines. The extracted metabolites were analyzed using a gas chromatograph system (Agilent) equipped with a Restek Rxi-5 ms capillary column. A sample volume (1 μL) was injected in split mode with injector temperature of 250 °C. The flow rate of helium used as a carrier gas was 1.1 mL/min. The initial temperature was held at 40 °C for 1 min, then ramped at 10 °C per min until 340 °C (hold time 10 min). Electron ionization (EI) was used with MS source temperature at 230 °C and Quad temperature at 150 °C. The metabolites were identified by comparison of the obtained spectra with those stored in the NIST mass spectra database.

### Statistical analysis

All experiments were carried out in triplicate and results were analyzed with the Minitab software version 19.2020.1 (Minitab Inc., US) and NCSS 2020 (NCSS, LIC, Utah, USA). The obtained data were analyzed statistically to determine the degree of significance using one-way analysis of variance (ANOVA) with Tukey–Kramer multiple comparisons and *t*-Student's tests at *p*-value ≤ 0.05.

## Supplementary Information

**Additional file 1**:** Table S1**. Performance of the constructed yeast consortium NYC-1 on decolorizing different azo dyes.** Table S2**. Hydrocarbon fractions of hexane extract of the AO7-degraded NYC-1 consortium as analysed by GC-MS for biodiesel production.** Table S3**. Physicochemical properties of biodiesel produced by AO7-degraded NYC-1 consortium.

## Data Availability

The datasets used and/or analyzed during the current study are available from the corresponding author on reasonable request.

## Abbreviations

TAG: Triacylglycerides
FAMEs: Fatty acid methyl esters
MUFA: Mono-unsaturated fatty acid
SFA: Saturated fatty acid
PUFA: Poly-unsaturated fatty acid
CN: Cetane number
IV: Iodine value
SV: Saponification value
*ν*: Kinematic viscosity
*ρ*: Density
OS: Oxidative stability
LCSF: Long-chain saturation factor
DU: Degree of unsaturation
DCW: Dry cell weight
AMP: Adenine-mono-phosphate
IMP: Inosine-mono-phosphate
ClO_2_: Chlorine dioxide
TCA: Tricarboxylic acid
Lac: Laccase
LiP: Lignin peroxidase
MnP: Manganese peroxidase
YPD: Yeast peptone dextrose
BH: Bushnell Hass
FAD: Flavin nucleotide
AO7: Acid Orange 7
RG19: Reactive Green 19
SGR: Scarlet GR
RB5: Reactive Black 5
MO: Methyl Orange
RB19: Reactive Blue 19
RB81: Reactive Blue 81
MR: Methyl Red
RR120: Reactive Red 120
RV5: Reactive Violet 5
