# Supplementary material for: Coupling azo dye degradation and biodiesel production by manganese-dependent peroxidase producing oleaginous yeasts isolated from wood-feeding termite gut symbionts
Source: Biotechnol Biofuels. 2021 Mar 8;14:61. doi: 10.1186/s13068-021-01906-0 (PMC7938474; doi:10.1186/s13068-021-01906-0)
Supplement: Supplementary file 1 — Additional file 1: Table S1. Performance of the constructed yeast consortium NYC-1 on decolorizing different azo dyes. Table S2. Hydrocarbon fractions of hexane extract of the AO7-degraded NYC-1 consortium as analysed by GC-MS for biodiesel production. Table S3. Physicochemical properties of biodiesel produced by AO7-degraded NYC-1 consortium. [file 13068_2021_1906_MOESM1_ESM.docx]

**Table S1**

Performance of the constructed yeast consortium NYC-1 on decolorizing different azo dyes.

| Azo dye | λ_max_ (nm) | Maximum decolorization (%) at a dye concentration of 250 mg/L | Time (h) required for maximum percentage decolorization |
| --- | --- | --- | --- |
|  |  |  |  |
| Acid Orange 7 (AO7) | 484 | 98.34 | 6 |
| Reactive Black 5 (RB5) | 595 | 81.45 | 18 |
| Reactive Blue 19 (RB19) | 592 | 87.67 | 15 |
| Reactive Red 120 (RR120) | 537 | 76.20 | 21 |
| Reactive Green 19 (RG19) | 630 | 75.46 | 18 |
| Methyl Orange (MO) | 465 | 90.45 | 15 |
| Methyl Red (MR) | 424 | 88.51 | 12 |
| Scarlet GR (SGR) | 511 | 95.32 | 12 |
| Reactive Violet 5 (RV5) | 530 | 75.21 | 18 |
| Reactive Blue 81 (RB81) | 581 | 83.0 | 24 |

**Table S2**

Hydrocarbon fractions of hexane extract of the AO7-degraded NYC-1 consortium as analysed by GC-MS for biodiesel production.

| Hydrocarbon | Name of hydrocarbon | Area (%) of FAME extract | Area (%) of hexane extract of dye treated consortium | Area (%) of hexane extract of non-treated dye consortium |
| --- | --- | --- | --- | --- |
| **Alkane** | n-undecane | 0.33 | 0.21 | ND |
|  | n-dodecane | 0.61 | 1.59 | ND |
|  | n-tetradecane | 0.25 | 0.62 | ND |
|  | n-pentadecane | 1.34 | 0.81 | ND |
|  | n-hexadecane | 2.73 | 0.35 | 0.45 |
|  | n-heptadecane | 2.34 | 0.31 | 2.25 |
|  | n-octadecane | 6.4 | 0.57 | 0.55 |
|  | n-nonadecane | 2.74 | 0.66 | 0.65 |
|  | n-eicosane | 8.2 | 0.70 | 6.32 |
|  | n-heneicosane | 1.73 | 4.02 | 1.93 |
|  | n-docasane | 2.32 | 0.73 | 1.02 |
|  | n-tetracosane | 1.22 | 0.12 | ND |
|  | n-hexacosane | 1.17 | 0.52 | ND |
|  | n-octacosane | 3.23 | 0.21 | ND |
|  | n-nonacosane | 0.80 | ND | ND |
|  | n-dotriacontane | 0.35 | 2.78 | ND |
|  | n-tetratriacontane | 1.24 | 2.96 | 1.03 |
|  | n-nonane | 0.62 | 0.11 | ND |
|  | n-decane | 1.54 | 0.32 | ND |
|  |  |  |  |  |
| **Alkene** | Hexadecene | 0.43 | 4.65 | 0.11 |
|  | Tetracosahexaene | 0.55 | 1.89 | ND |
|  |  |  |  |  |
| **SFA** | Butanoic acid | 0.34 | 0.29 | ND |
|  | Pentanoic acid | 0.53 | 0.79 | ND |
|  | Hexanoic acid | 0.12 | ND | ND |
|  | Heptenoic acid | 0.42 | ND | ND |
|  | Tridecanoic acid | ND | 0.42 | ND |
|  | Tetradecanoic acid | 1.12 | 0.83 | ND |
|  | Dodecanoic acid | 1.79 | 0.44 | 55.18 |
|  | Hexadecanoic acid | 0.62 | 0.41 | 2.83 |
|  | Octadecanoic acid | 0.80 | 2.87 | 1.64 |
|  | Eicosanoic acid | 0.93 | 0.52 | ND |
|  | Docasanoic acid | 0.55 | 0.98 | ND |
|  | Tetracosanoic acid | 1.34 | 0.62 | ND |
|  |  |  |  |  |
| **USFA** | Nonenoic acid | ND | ND | ND |
|  | Nonadecenoic acid | 0.18 | 0.11 | ND |

**SFA**, saturated fatty acid; **USFA**, unsaturated fatty acid; **ND**, not determined.

**Table S3**

Physicochemical properties of biodiesel produced by AO7-degraded NYC-1 consortium.

| Biodiesel properties | International diesel standard | International biodiesel standard-ASTM (US) | NYC-1 consortium |
| --- | --- | --- | --- |
| Cetane number | 45 | Min 47 | 53 |
| Density (g/cc) | 0.838 | 0.86-0.9 | 0.841 |
| Kinematic viscosity (mm^2^/s) | 1.9 – 4.1 | 1.9 – 6.0 | 4.38 |
| Acid value (mg of NaOH/g of oil) | Max 0.5 | Max 0.8 | 0.273 |
| Distillation temperature | 372 °C | 360 °C | 364 °C |
